# Assessment of mTOR-Dependent Translational Regulation of Interferon Stimulated Genes

**DOI:** 10.1371/journal.pone.0133482

**Published:** 2015-07-24

**Authors:** Mark Livingstone, Kristina Sikström, Philippe A. Robert, Gilles Uzé, Ola Larsson, Sandra Pellegrini

**Affiliations:** 1 Cytokine Signaling Unit, Institut Pasteur, CNRS URA1961, Paris, France; 2 Department of Oncology-Pathology, Karolinska Institutet, Stockholm, Sweden; 3 CNRS UMR5235, University of Montpellier II, Montpellier, France; Institut national de la santé et de la recherche médicale - Institut Cochin, FRANCE

## Abstract

Type-I interferon (IFN)-induced activation of the mammalian target of rapamycin (mTOR) signaling pathway has been implicated in translational control of mRNAs encoding interferon-stimulated genes (ISGs). However, mTOR-sensitive translatomes commonly include mRNAs with a 5’ terminal oligopyrimidine tract (TOP), such as those encoding ribosomal proteins, but not ISGs. Because these translatomes were obtained under conditions when ISG expression is not induced, we examined the mTOR-sensitive translatome in human WISH cells stimulated with IFN β. The mTOR inhibitor Torin1 resulted in a repression of global protein synthesis, including that of ISG products, and translation of all but 3 ISG mRNAs (*TLR3*, *NT5C3A*, and *RNF19B)* was not selectively more sensitive to mTOR inhibition. Detailed studies of *NT5C3A* revealed an IFN-induced change in transcription start site resulting in a switch from a non-TOP to a TOP-like transcript variant and mTOR sensitive translation. Thus, we show that, in the cell model used, translation of the vast majority of ISG mRNAs is not selectively sensitive to mTOR activity and describe an uncharacterized mechanism wherein the 5’-UTR of an mRNA is altered in response to a cytokine, resulting in a shift from mTOR-insensitive to mTOR-sensitive translation.

## Introduction

Upon type-I interferon (IFN) binding to its dimeric receptor IFNAR1:IFNAR2, the associated Janus kinases, Jak1 and Tyk2, are activated and phosphorylate IFNAR2 on tyrosine (Tyr) residues [1]. These phosphorylation sites serve as docking sites for Stat transcription factors, facilitating their phosphorylation by Jaks. Tyrosine phosphorylated Stat1 and Stat2 associate with IRF9 to form the transcriptionally active IFN-stimulated gene factor-3 (ISGF3) complex that induces transcription of interferon-stimulated genes (ISGs) via interferon-stimulated response elements (ISRE) in promoters of target genes [2]. ISGs include not only those genes whose transcription is activated by ISGF3, but also those whose regulation depends on transcription factors that are themselves encoded by ISGs (*e*.*g*. IRF1) [3]. A number of reports have demonstrated cross-talk activation of non-Jak/Stat signal transduction pathways by IFN in a variety of cell lines [4–15]. In particular, pharmacological inhibition, genetic knockout, and small interfering RNA (siRNA) knockdown have demonstrated that canonical PI3K-mTOR pathway components are also required for IFN-induced cross-talk signaling [5]. Moreover, these studies have demonstrated that altered murine ISG protein levels (*e*.*g*. *Isg15*, *Cxcl10*, *Irf7*, *Ifit2*, *Slfn2*) observed in mTOR pathway knockout cells after IFN stimulation are not the result of altered transcription, but rather translation. A model was therefore suggested where IFN binding to its receptor leads to cross-talk at the level of IRS1 which mediates activation of the mTOR pathway and thereby selective increased translation of ISG mRNAs. Such a mechanism would allow cells to mount an anti-viral response even when nutrients are insufficient for high rates of total protein synthesis. However an RNA element or RNA binding protein mediating such selective activation of ISG translation has not been identified.

The mammalian target of rapamycin (mTOR) is an atypical serine/threonine kinase named for its sensitivity to the naturally occurring compound rapamycin, which is clinically approved for immunosuppressive and anticancer therapy [16]. Signals from growth factors and nutrient availability converge on mTOR resulting in modulation of anabolic vs. catabolic processes, including growth, proliferation, autophagy and mitochondrial biogenesis [17,18]. Some of the functions of mTOR are a result of its ability to modulate translational efficiency. Although the rapamycin-sensitive phosphoproteome contains many direct mTOR substrates [19], the best characterized targets that affect translation are the 70 kDa ribosomal protein S6 kinase (p70S6K) and the eukaryotic [translation] initiation factor 4E (eIF4E)-binding protein 1 (4E-BP1). While p70S6K phosphorylation at Thr389 is required for its activity and is blocked by rapamycin, 4E-BP1 is phosphorylated on multiple sites by mTOR, and it is the largely rapamycin-insensitive phosphorylation of Thr46 that prevents inhibitory binding to the 5’ mRNA cap-binding protein eIF4E [20,21]. A proposed role of the rapamycin-sensitive Ser65 in this binding has been the subject of controversy [22–26]. Rapamycin-insensitive mTOR activities can be inhibited with ATP competitive inhibitors, including Torin1, that directly target the kinase domain [27]. Downstream of mTOR the translation of certain mRNAs with 5’ terminal tracts of oligopyrimidines (TOP) encoding *e*.*g*. ribosomal proteins are particularly reliant on mTOR. The ability of mTOR to selectively regulate TOP mRNA translation is independent of S6 ribosomal protein (RPS6) phosphorylation and 4E-BP1 under most physiological conditions [28–30]. Multiple culprit proteins have been implicated in the regulation of TOP mRNA translation downstream of mTOR, most recently LARP1 [31].

In previous gene expression microarray and ribosome profiling studies aimed at identifying human and murine mRNAs translated less efficiently upon mTOR inhibition, most well-characterized ISGs are conspicuously absent among regulated genes [32–36]. Instead, these studies identified subsets of mRNAs containing TOP motifs, TOP-like motifs, and/or pyrimidine-rich translational elements (PRTEs) within their 5’ untranslated regions (UTRs). TOP mRNAs are defined as those with a cytidine at their 5’ end immediately followed by 5 to 15 pyrimidines, while TOP-like mRNAs must only have a stretch of pyrimidines within 4 bases of the mRNA cap [36]. Subsequent analyses indicated that PRTEs appear to not be enriched in mTOR target mRNAs [37]. As these studies did not include IFN treatment, it is possible that many ISGs were not sufficiently abundant to allow assessment of their translational efficiency. Additionally, the impact of mTOR inhibition on mRNA translation could be different after IFN-induced physiological changes occur. Therefore we evaluated the ability of mTOR inhibitors to selectively repress the translation of ISG mRNAs during an IFN treatment. Through this approach we are able to assess directly whether IFN-induced mRNAs are relatively more dependent on the mTOR pathway for their translation. Human WISH cells have been widely used as a model system of IFN studies and were chosen here for their well-characterized IFN response and the quality of polysome profiles generated.

## Materials and Methods

### Cell lines and reagents

WISH cells were cultured as previously described [38,39]. WISH cells were obtained from G. Uzé (CNRS, Montpellier) and originate from ATCC (CCL-25). Their identity was compared to HeLa S3 cells by short tandem repeat (STR) loci profiling. This analysis, performed by IdentiCell (Department of Molecular Medicine, Aarhus University Hospital), showed that the two cell lines have identical STR profiles. Additional cell lines were kindly provided by: O. Acuto (Daudi and Jurkat cells), E. Ségal-Bendirdjian (NB4 cells), C. Bono and B. Arnulf (U266 cells), E. Coccia (Molt-4), and maintained in RPMI and 10% heat-inactivated fetal calf serum. Suspension cells were routinely subcultured at 5 × 10^4^ cells/ml. Recombinant IFN α2 was from D. Gewert (Wellcome, UK); IFN β was from Biogen Idec (Boston, MA). IFNs were purified to specific activities > 10^8^ IU/mg of protein. Rapamycin, cycloheximide and Triton X100 were purchased from Sigma, sodium deoxycholate was from Fluka, and Torin1 was from Tocris.

### Isolation of polyribosome-associated mRNA

WISH cells were trypsinized and plated in 10cm tissue culture dishes at 4 X 10^6^ cells/dish. After 30 hrs, cells were re-fed with fresh medium with or without IFN β (100pM) and/or mTOR inhibitors at saturating concentrations (rapamycin 100nM or Torin1 1μM). After 12 hrs, cells were pretreated with cycloheximide (100μg/ml, 15 min) and washed with cold phosphate-buffered saline (PBS) containing cycloheximide (100μg/ml). The intact cells were scraped in 425μl hypotonic lysis buffer (5 mM Tris-HCl [pH 7.5], 2.5 mM MgCl_2_, 1.5 mM KCl). The lysates were incubated with 100 μg/ml cycloheximide, 2 mM dithiothreitol (DTT), and 5μl rRNasin (Promega 29457913), kept on ice for 5 min and vortexed. Triton X-100 (0.5%) and sodium deoxycholate (0.5%) were added to each sample. Following another round of vortexing, samples were incubated on ice (5 min). Cell extracts were subjected to microcentrifugation for 5 min at 14,000g at 4°C, and supernatants were collected. Two-thirds of each lysate was loaded onto pre-chilled sucrose gradients (10%-50% sucrose, 200mM HEPES pH 7.6, 1M KCl, 50mM MgCl_2_) in a Beckman centrifuge tubes (14 by 89 mm) (catalog no. 331372; Beckman Instruments) and the remaining third was saved for isolation of total cytoplasmic RNA fraction. The tubes were then centrifuged in a Beckman SW41Ti rotor at 36,000 rpm for 3 h at 4°C. Fractions were collected per sample using a Buchler Isodensiflow II fractionator and the 254 nm absorbance was measured. The optical density 254 nm data were acquired using an analog-to-digital converter device and PolysomeRider software. Fractions collected after the second local optical density peak following the 80S peak were considered polyribosome-associated, collected, and immediately mixed with TRIzol (Invitrogen).

### Quantitative real time PCR (qPCR)

TRIzol (Invitrogen)-isolated RNA was cleaned using RNeasy Mini Kit (Qiagen 74104) according to manufacturer's instructions including RNase-Free DNase Set (Qiagen 79254) on column DNase digestion. RNA abundance and quality was assessed by Nanodrop and Agilent Bioanalyzer, respectively. Reverse Transcription was performed using Moloney murine leukemia virus (M-MLV) Reverse Transcriptase (Invitrogen 28025–013) according to manufacturer's instructions with Random Primers (Invitrogen 58875) and rRNasin (Promega 29457913). qPCR was performed as previously described [38]. Briefly, cDNA was purified with QIAquick PCR Purification Kit (Qiagen 28104). qPCR was performed with FastStart Universal SYBR Green Master Mix (Roche 4913850001) and StepOne Plus Machine (Applied Biosystems) using standard curve-based quantification with 60°C annealing temperature and three technical replicates. PCR product standards were produced as above, purified with QIAquick PCR Purification Kit and diluted in Tris-EDTA (TE) Buffer with 10 μg/ml sheared salmon testes DNA (Sigma D-9156). PCR products for each primer pair were subjected to sequencing. The forward and reverse primers were used for qPCR analyses of gene expression are presented in S1 and S2 Tables.

### Genome-wide microarray

Genome-wide mRNA expression was assessed using Human Gene 1.1 ST 16-Array Plates (Affymetrix 901417) and the GeneTitan instrument (n = 4 biological replicates). Data were normalized and summarized using robust multiarray averaging (RMA) in combination with updated probe-set definitions as these improve the precision and accuracy of Affymetrix DNA microarrays [40,41]. The resulting data were examined using principal components analysis and replicate experiment clustered according to biological condition indicating good reproducibility. Because changes in levels of polysome-associated mRNA can be the result of changes in levels of cytosolic mRNA, differences in polysome-associated mRNA need to be adjusted for differences in cytosolic mRNA levels to identify differential translation. Herein, differential translation was identified using the anota R package which shows improved performance as compared to commonly used translation efficiency (TE) scores in as much as differential translation identified by anota but not TE scores correlates to changes in protein levels [42–44]. Anota was applied using the following settings which includes a false discovery rate (FDR) threshold of 0.15: minSlope = (-1); maxSlope = 2; slopeP = 0.01; maxRvmPAdj = 0.15; selDeltaPT = log2(1.5). Anota uses a random variance model (RVM) to identify differential translation and changes in cytosolic mRNA levels were also identified using RVM [45]. We selected a relaxed FDR threshold (15%) to make sure that we did not miss ISGs as showing differential translation due to a too strict threshold. Data are available at the gene expression omnibus (GEO) with accession GSE70270.

### Transcriptional start site determination

mRNAs purified as for qPCR and with a 5’ cap and a poly(A) tail were subject to rapid amplification of cDNA ends (RACE) including oligocapping and poly(A) tailing with additional oligonucleotide sequences using a commercially-available kit (ExactSTART Eukaryotic mRNA 5’- & 3’-RACE Kit, Epicentre, ES80910). cDNA was purified with RNeasy Kit (Qiagen) after the alkaline phosphatase treatment step. PCR was performed using Q5 Hot Start High-Fidelity DNA Polymerase (NEB M0493) according to manufacturer's instructions. Reverse primers (S1 Table) designed to give products of 300–1000 bp in length were used for a subsequent round of PCR in combination with the oligo-capped Primer 1 (Epicentre kit). After another round of PCR using oligo-capped Primer 1 and one of 12 different nested reverse primers (S1 Table), visible (EtBr stained agarose gel) PCR products were purified (QIAquick Gel Extraction Kit, Qiagen 28704), and subjected to Sanger sequencing (Eurofins).

### Western blot analysis

As with isolation of polysome-associated mRNA, WISH cells were trypsinized and seeded in 10 cm tissue culture dishes at 4 x 10^6^ cells/dish. After 30 hrs, cells were re-fed with fresh medium with or without IFN β (100pM) and/or mTOR inhibitors (rapamycin 100nM or Torin1 1μM) for 12 hrs. For signaling studies in serum-starvation conditions, WISH cells were trypsinized and plated as described above before undergoing a 24-hr serum-starvation prior to treatment with serum or IFN. The cells growing in suspension (Daudi, Jurkat, NB4, and U266) were subjected to a 4-hr serum starvation prior to treatment with serum or IFN. All cells were lysed in RIPA buffer with protease and phosphatase inhibitors as described previously [39] with the addition of a probe sonication step. Antibodies used were against USP18, Akt RMAB (a pan-AKT), P-Akt Thr308, P-Akt Ser473, P-4E-BP1 Ser65, 4E-BP1, P-ERK1/2 Thr202/Tyr404, ERK1/2, RPL26, TLR3, S6K1, P-S6K1 Thr389, eIF4B, P-eIF4B Ser422, RPS6, P-RPS6 Ser235/236, P-RPS6 Ser240/244 (Cell Signaling Technology), Stat1 (Millipore), Stat2 (UBI), Actin (Sigma), PKR (Agro-bio), ASPH, CD274 (Abnova), HIST1H1B, NT5C3A, Akt mAb (Santa Cruz) that detects only AKT1, IFIT1/ISG56, IFIT3/ISG60 (gifts from G. C. Sen), OAS2 p69 (a gift from A.G. Hovanessian), and ISG15 (a gift from E. Borden). Immunoblots were revealed using enhanced chemiluminescence detection reagents (Western Lightning, PerkinElmer). All western blot images were analyzed with ImageJ prior to use in figures to prevent the use of saturated images.

## Results

### IFN fails to activate the mTOR signaling pathway in many cell lines

**Fig 1:**
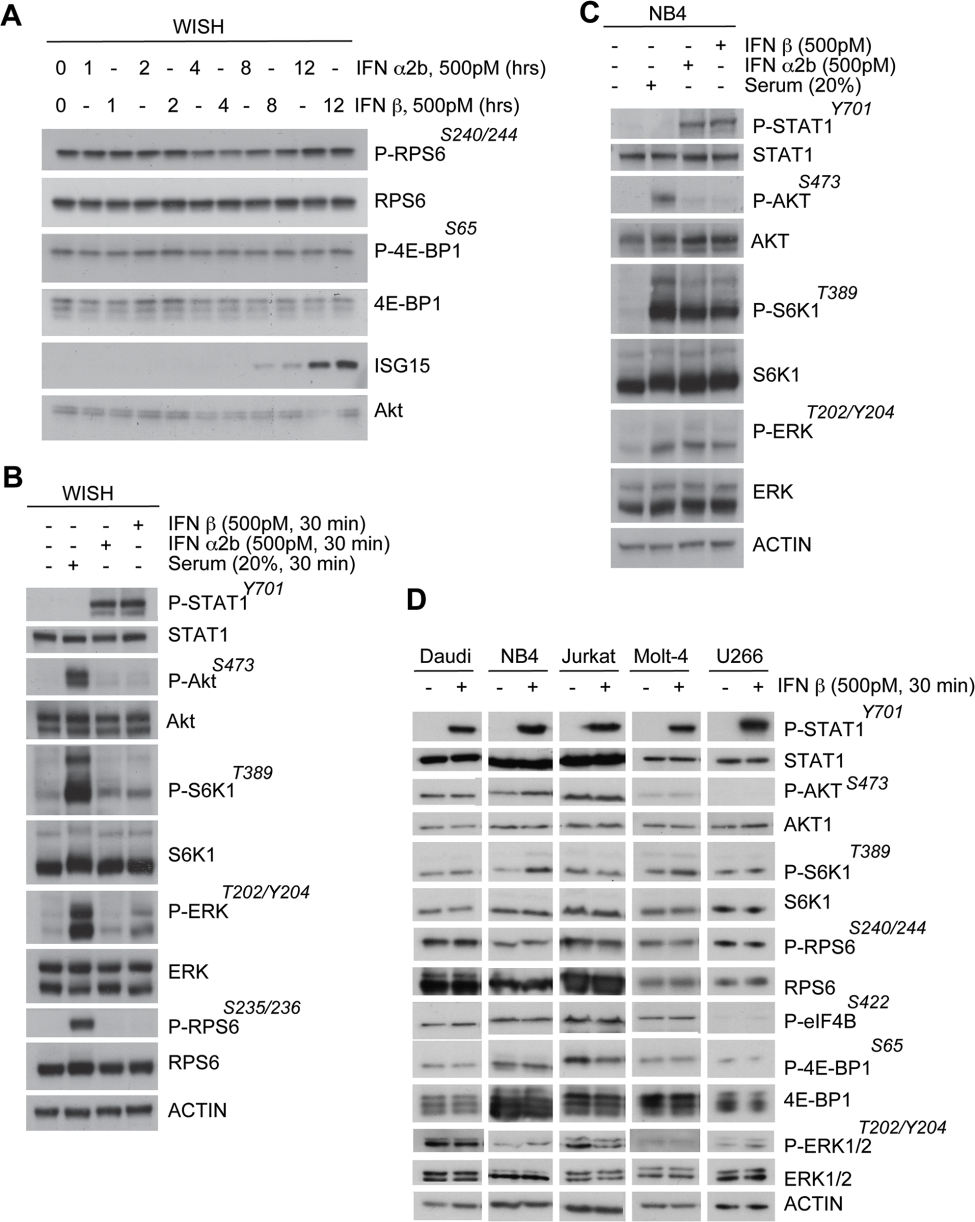
Type-I IFN fails to activate the Akt-mTOR pathway in the majority of cell lines tested. (A) IFN α2b and IFN β, added for various times to WISH cells cultured in 10% serum, fail to significantly alter 4E-BP1 phosphorylation at Ser65. (B) Western blot analyses of the activation of the Akt-mTOR and ERK pathways in WISH cells subjected to serum starvation for 24 hrs prior to IFN or serum stimulation. (C) Western blot analyses of the activation of the Akt-mTOR and ERK pathways in NB4 cells subjected to serum starvation for 24 hrs prior to IFN or serum stimulation. (D) Western blot analyses comparing activation of the Akt-mTOR and ERK pathways in Daudi, NB4, Jurkat, Molt-4, and U266 cells subjected to serum starvation for 4 hrs prior to IFN β stimulation.

To assess the impact of IFN on the mTOR pathway, WISH cells were subjected to treatment with IFN (subtype α2b or β) for various times (1, 2, 4, 8, and 12 hrs). Western blot results failed to demonstrate a reproducible modulation in mTOR signaling to 4E-BP1 or RPS6 (Fig 1A). As these experiments were performed in complete medium (10% serum) with relatively high nutrient sensing signaling, potential acute Akt-mTOR pathway activation by IFN (500pM, 30 min) was studied in WISH cells subjected to 24-hr serum starvation. Similarly, this analysis did not identify changes in phosphorylation of mTOR substrates depending on IFN treatment (Fig 1B). Other human cell lines (Jurkat, NB-4, Daudi, Molt-4, and U266) were also evaluated for induction of the Akt-mTOR pathway in response to IFN (Fig 1C and 1D). Although minor lane-to-lane variations in band intensity were observed, with the exception of p70S6K Thr389 phosphorylation evident in NB4 cells, no IFN-induced phosphorylation of p70S6K, Akt, 4E-BP1 or RPS6 could be reproducibly detected. Thus, in five of the six cell cell lines analyzed, IFN does not appear to affect mTOR signaling.

### Reduced ISG protein levels following mTOR inhibition is consistent with the global effects on translation

**Fig 2:**
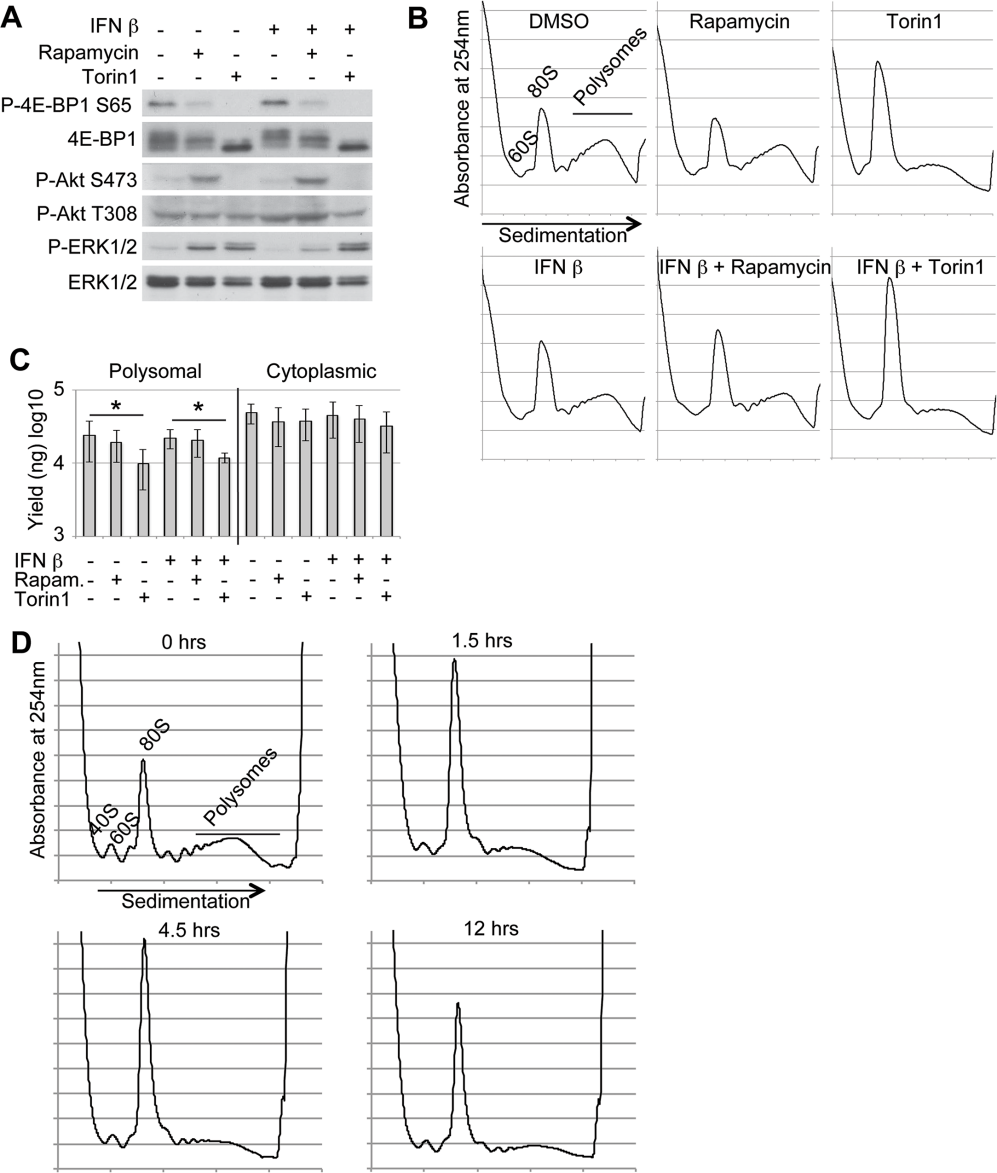
The mTOR inhibitor Torin1 results in a global reduction of mRNA translation, including of ISG mRNAs during IFN treatment. (A) WISH cells treated for 12 hrs with DMSO (-), 100nM rapamycin or 1μM Torin1 alone or in combination with IFN β (100pM) were subjected to western blot to assess phosphorylation of the indicated proteins. (B) Polysome profiles of WISH cells treated as in (A). (C) RNA abundance in nanograms (ng) as quantified by Nanodrop to determine yields of isolated RNA from cytoplasmic and polyribosome-associated (polysomal) fractions of WISH cells treated with DMSO (-), 100nM rapamycin, or 1μM Torin1 alone or in combination with IFN β (100pM) for 12 hrs. Shown are means +/- standard deviations (n = 7 biological replicates). * indicates p-values < 0.05. (D) Polysome profiles of WISH cells treated with Torin1 (1μM) for 0, 1.5, 4.5, or 12 hrs.

To study the effect of mTOR inhibition on ISG mRNA translation, we used conditions of optimized ISG expression. We have previously observed that many commonly studied ISG mRNAs reach their maximal levels after 12 hrs of IFN stimulation and that IFN β produces a more sustained IFN response than does IFN α in WISH cells [38]. Western blot analyses of WISH cell lysates revealed the expected signaling pattern in response to mTOR inhibitors (Fig 2A). In accordance with previous reports, Torin1 more strongly reduced phosphorylation of 4E-BP1 than did rapamycin. Moreover, Torin1 reduced, while rapamycin enhanced, Akt phosphorylation at the mTOR complex 2 (mTORC2) phosphorylation site, Ser473, but not at the phosphatidylinositide-dependent kinase 1 (PDK1) site Thr308. mTOR inhibition also resulted in an increase of ERK1/2 Thr202/Tyr204 phosphorylation, consistent with previous reports [46,47]. Again, no significant increase in 4E-BP1, Akt, or ERK1/2 phosphorylation was observed in WISH cells subjected to this 12 hrs-IFN β treatment in the presence of 10% serum (Fig 2A). Thus, these conditions were used to study the effect of inhibition of the mTOR pathway on ISG mRNA translation.

Polysome-preparations can be used to monitor global effects on translation by comparing RNA amounts in polysomes to the amount of free 80S ribosomes. To assess such global effects on translation in our experimental setting, we isolated polysome-associated mRNA (*i*.*e*. mRNAs bound by more than 3 ribosomes) from WISH cells untreated or treated with mTOR inhibitors and/or IFN from seven independent experiments (one representative tracing from each condition is shown in Fig 2B). Previous work demonstrated that kinase inhibitors targeting both mTORC1 and mTORC2 can inhibit global translation rates considerably (approximately 50%), while rapamycin has only a modest effect [36,48]. Consistently, Torin1 treatment for 12 hrs, alone or in combination with IFN β led to an increase in the 80S peak and a concomitant decrease in polysome-associated mRNA (Fig 2B). Quantification of the amounts of recoverable polysome-associated mRNA under the conditions used corroborated that Torin1 reduced the amount of such RNA by 40–45% (Fig 2C). Such a change was evident already after a 1.5 hr-treatment with Torin1 (Fig 2D). While these findings were consistent with a global reduction in translation in response to Torin1 treatment, they could also indicate suppressed translation of a subset of highly expressed mRNAs. Western blot conditions were optimized for assessing the level of ISG-encoded proteins in the absence and presence of mTOR inhibitors. Since Torin1 may repress translation, induce autophagy and reduce proliferation, which all affect total amount of protein, an equal number of cells were seeded and equal volumes of lysate were analyzed by Western blots. Indeed the level of actin was reduced approximately 25% in the Torin1-treated sample (S1A Fig). The levels of 11 ISG-encoded proteins were similarly reduced in cells treated with IFN in the presence of Torin1. Consistent with an approximate 50% reduction in protein synthesis in the Torin1-treated sample, analysis of twice the quantity of lysate revealed levels of ISG-encoded proteins comparable to those in the lysate treated with IFN alone (S1A Fig).

This 50% reduction may be caused by reduced proliferation, increased autophagy, reduced translation, and/or other cellular phenomena. Thus, the effect on ISG protein levels following Torin1 treatment is consistent with the observed global repression of translation. Nevertheless, there may be subsets of mRNAs encoding ISGs showing selectively suppressed translation.

### Identification of mRNAs reliant on mTOR for their translation in IFN-stimulated WISH cells

**Fig 3:**
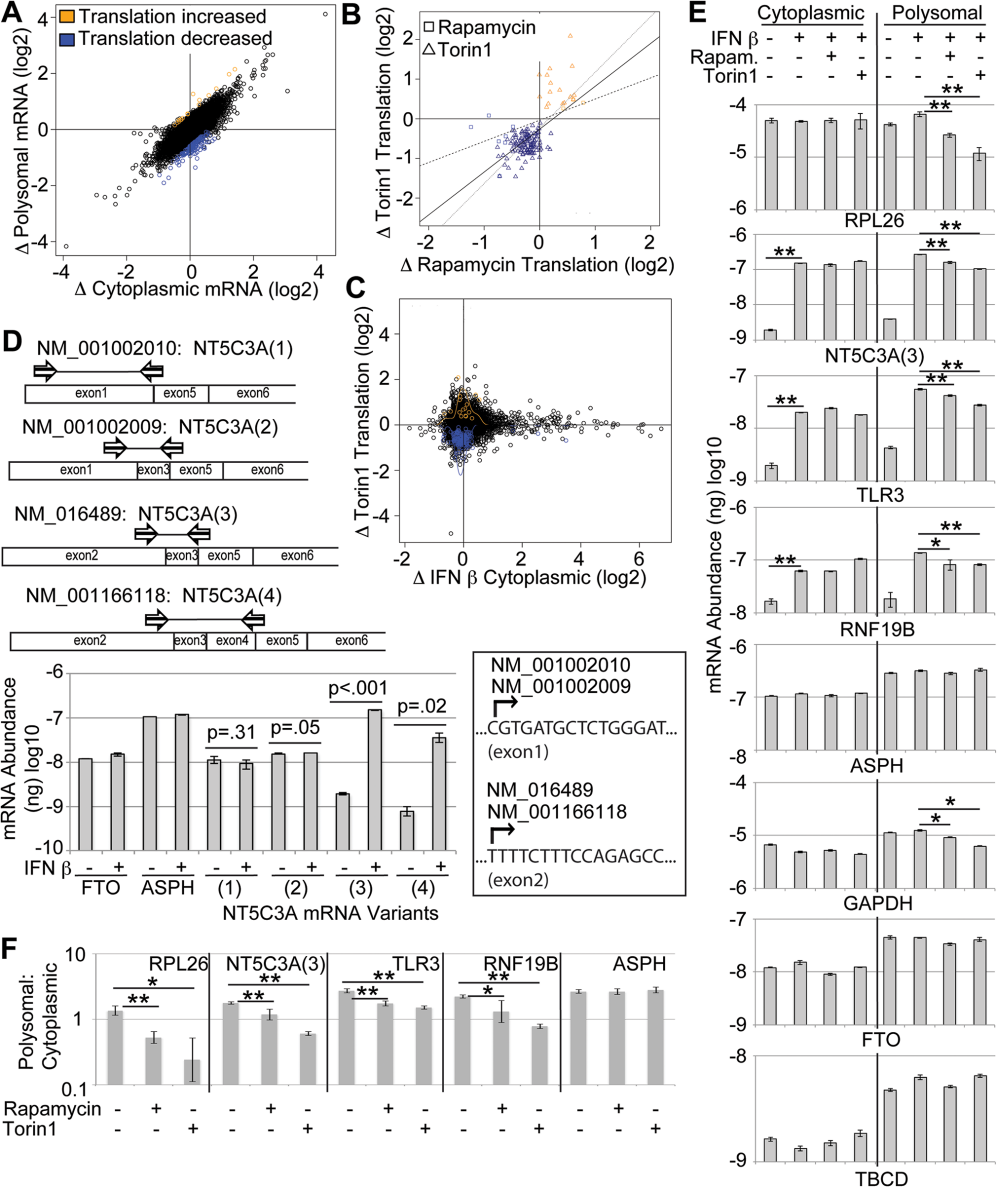
Polysome-profiling to identify ISG mRNAs subject to mTOR-dependent translational control. (A) RNA isolated from WISH cells treated with IFN β (100pM) in combination with DMSO or 1μM Torin1 for 12 hrs was subjected to comparative genome-wide mRNA expression profiling, and genes showing differential translation were identified using anota. Genes are plotted according to changes (Δ) in cytoplasmic and polysomal mRNA levels upon the addition of Torin1 to IFN β-treated cells. Genes showing repressed (blue) and enhanced (orange) mRNA translation in response to Torin1 are indicated. (B) Correlation between Torin1 (square)- and rapamycin (triangle)-induced mRNA translation changes (Δ) in IFN β-treated cells. Genes identified in either comparison that show increased (yellow) or decreased (blue) translation are indicated by condition where they were identified (squares or triangles). (C) A lack of correlation is observed when Torin1-induced mRNA translation changes (y-axis) and IFN β-induced cytoplasmic mRNA changes (x-axis) are plotted for all assessed genes. (D) *NT5C3A* mRNA variants 3 and 4 are transcriptionally induced by IFN. Schematic representation of *NT5C3A* mRNA variant-specific PCR primers used for qPCR. Arrows represent forward and reverse primers. mRNA abundance in nanograms (ng) was assessed by quantitative polymerase chain reaction (qPCR) for the indicated genes, including mRNA variants 1–4 of *NT5C3A*. Shown are means +/- standard deviations (n = 3). Theoretical transcriptional start sites (arrows) based on NCBI reference sequences are shown (box). (E) Cytoplasmic and polysomal mRNA abundance (ng) was assessed by qPCR for the indicated genes. Shown are means +/- standard deviations (n = 3). * indicates p-values < 0.05 and ** < 0.005. (F) Polysomal-to-cytoplasmic mRNA ratios were calculated (Student’s t-test) for select genes in **e** and are shown as means and 95% confidence intervals. * indicates p-values < 0.05 and ** < 0.005.

Next we aimed to assess if translation of a subset of ISGs is selectively modulated following mTOR inhibition. A common approach to study translation genome-wide involves isolation of polysome-associated mRNA followed by their quantification using DNA microarrays or RNA-seq [49]. In contrast to ribosome profiling, this method allows for quantification of only those mRNAs that are efficiently translated (*e*.*g*. mRNAs associated with >3 ribosomes) [50]. Importantly, regulation of gene expression upstream of translation affecting levels of cytosolic mRNA will lead to changes in polysome-associated mRNA also in the absence of differential translation. Therefore, changes in polysome-associated mRNA between conditions need to be adjusted for corresponding changes in cytosolic mRNA levels to identify differential translation. To allow such adjustment, cytosolic mRNA levels are measured in parallel. Accordingly, to assess if translation of a subset of ISGs was modulated following mTOR inhibition, genome-wide expression analysis of cytoplasmic and polyribosome-associated fractions was performed. Because an equal amount of RNA was used as input from all conditions, this experiment reveals which mRNAs are relatively more or less affected than the average mRNA. When IFN β-treated cells were compared to cells treated with both Torin1 and IFN β, 139 mRNAs exhibited significantly reduced translation as identified using anota analysis (see Materials and methods, Fig 3A and S2 Table). By comparison, few mRNAs exhibited statistically significant changes in translation efficiency in response to rapamycin treatment during IFN stimulation (S2 Table). This is in accordance with previous work demonstrating that a relatively small subset of mRNAs shows significantly reduced translation efficiency in response to both rapamycin and active site mTOR inhibitors [35]. Nevertheless, trends for negative changes in translation efficiency were observed in response to rapamycin treatment for the majority of the 139 mRNAs exhibiting significant reduction in translation in response to Torin1 (Fig 3B). This finding is consistent with previous work demonstrating only partially inhibited translation of TOP mRNA by rapamycin [51]. Many of the mRNAs translationally downregulated by mTOR inhibition with Torin1 in our study were previously described to be mTOR translational targets [33,34], and 50 possessed a TOP or TOP-like motif (S3 Table). While only 17 mRNAs appeared to be TOP mRNAs according to their NCBI sequences, an analysis of predominant TSSs in normal adult tissues in the dbTSS [52], revealed TOP and TOP-like motifs present in 33 additional mRNAs. Overall, this analysis demonstrates that during IFN treatment a sustained inhibition of mTOR represses the translation of many mRNAs with TOP and TOP-like motifs at their 5’-end.

### With a few exceptions ISG mRNAs are not selectively reliant on mTOR for their translation

A genome-wide comparison of cytoplasmic mRNA levels between control and IFN β-treated cells identified 502 ISG mRNAs exhibiting increased expression (at least 1.5 fold) in response to IFN (S4 Table). The majority of the most highly induced genes are previously described ISGs, and only one (*i*.*e*. *CD68*) of the 100 most induced (ranked by fold-change) mRNAs could not be found in the Interferome database of ISGs identified in previous microarray gene expression studies [53]. Single-gene qPCR was performed and confirmed IFN-induced expression for all 97 genes tested, including *CD68* (S4 Table). When fold-induction of cytoplasmic mRNA by IFN was plotted against Torin1-induced fold-changes in translation efficiency a lack of correlation was observed (Fig 3C), demonstrating that the subset of ISG-encoded mRNAs is not enriched for mRNAs that are particularly reliant on mTOR activity for their translation. Of the 139 genes we identified as exhibiting significantly reduced mRNA translation in response to Torin1 treatment, only three (*NT5C3A*, *TLR3* and *RNF19B*) were among the 502 identified ISGs, indicating that ISG mRNAs are not more likely to be mTOR translational targets than non-ISG mRNAs (0.85 fold enrichment; p = 1). We next aimed to validate the regulation of these genes.

### The ISGs *TLR3*, *NT5C3A(3)* and *RNF19B* encode mRNAs exhibiting selectively decreased translation efficiency upon mTOR inhibition

The importance of proper reference gene selection has been highlighted in many qPCR studies, and the use of commonly used genes (*ACTB*, *GAPDH*, etc.) has been subject of criticism, leading some authors to propose the use of alternative genes, including ribosomal protein genes, for this purpose [54]. Indeed, it was suggested that the translation of *GAPDH* and *ACTB* mRNAs is suppressed in response to mTOR inhibition [33,34]. We also observed reduced levels of polyribosome-associated *GAPDH* mRNA in response to mTOR inhibitors (Fig 3E). The NCBI database *GAPDH* mRNA sequence is not TOP-like, but the predominant TSS in the dbTSS does yield an mRNA with a TOP-like sequence: 5’-GCTCTCTGCTCCTCCTG. Although we found cytoplasmic levels of ribosomal protein mRNAs to be relatively stable in our system (Fig 3E and data not shown), their translational repression in response to mTOR inhibition makes them inappropriate reference genes for polysome-associated mRNA levels. To identify potential reference genes in our system, we considered candidate genes predicted by our genome-wide microarray results to exhibit no change in expression in response to IFN and no change in translation efficiency in response to rapamycin or Torin1. Of the candidates, only PCR primers amplifying segments of *FTO* and *ASPH* cDNAs yielded PCR products. qPCR was performed for *ASPH*, *FTO*, *GAPDH*, and two other genes (*MSRP24* and *TBCD*) previously used as reference genes in an mTOR inhibition-mRNA translation study [35] (Fig 3E and data not shown). Of these, *ASPH*, *TBCD*, and *FTO* were found to be the most stable reference genes and were used for the subsequent analyses.

We first validated the selective mTOR-dependent translation of *NT5C3A*, *TLR3* and *RNF19B*. Four distinct mRNA variants arising from the *NT5C3A* gene are listed in the NCBI database and only one isoform was reported to be induced by IFN based on experiments in Raji cells [55,56]. We evaluated the four mRNA variants by qPCR and found that two variants (NM_016489 and NM_001166118) are transcriptionally induced by IFN, while two other variants (NM_001002010 and NM_001002009) are not (Fig 3D). The two IFN-stimulated mRNA variants arise from a distinct TSS in exon 2, which becomes the major TSS after IFN treatment and yields a TOP-like mRNA (Fig 3D box). qPCR was performed on equal ng quantities of RNA as input and confirmed that *TLR3*, *RNF19B*, and the major IFN-induced *NT5C3A* variant, *NT5C3A(3)*, are ISGs with reduced polyribosome-associated mRNA levels upon the inhibition of mTOR with rapamycin or Torin1 (Fig 3E and 3F).

**Fig 4:**
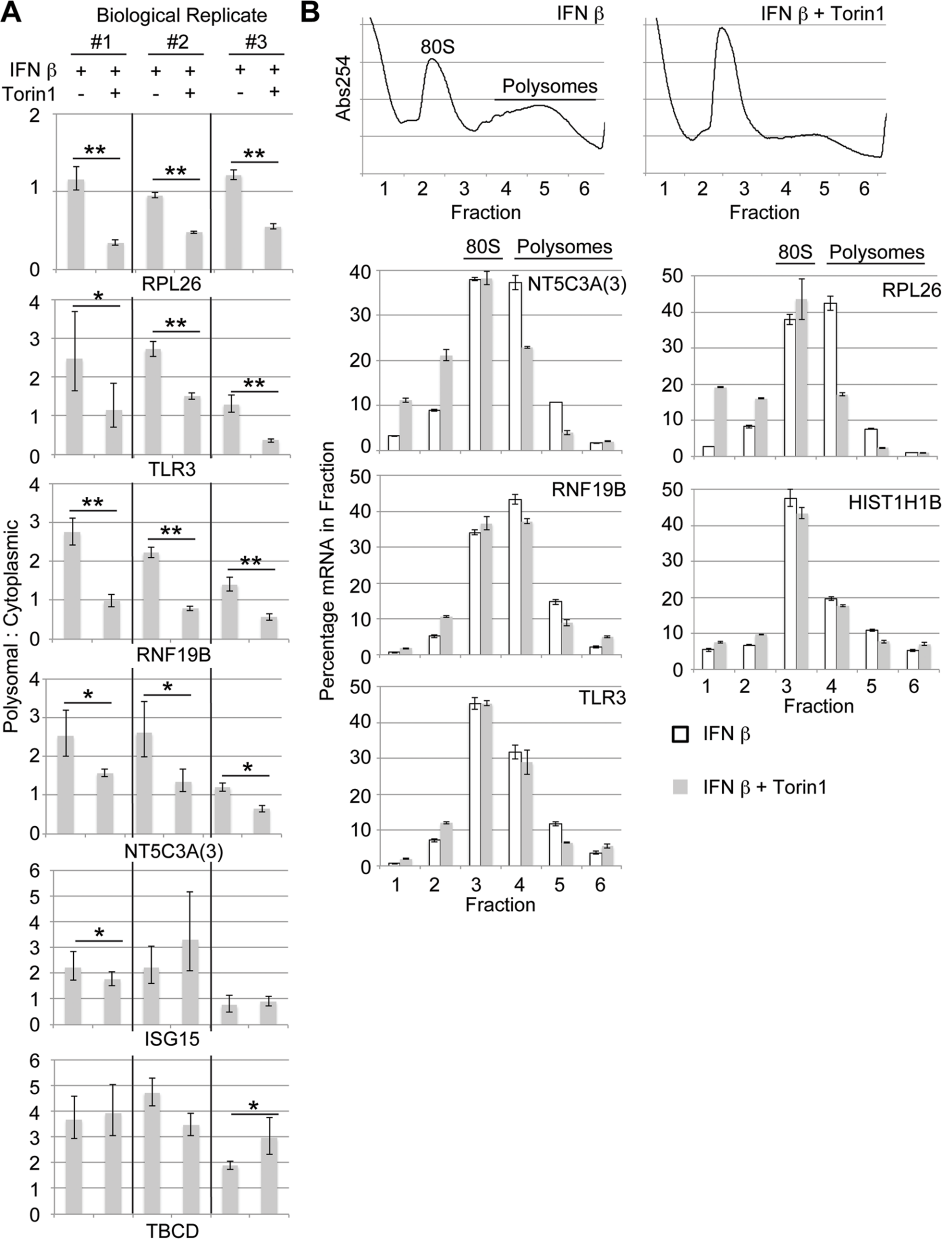
The ISGs *TLR3*, *NT5C3A*, and *RNF19B* encode mRNAs less efficiently translated during mTOR inhibition. (A) Polysomal-to-cytoplasmic mRNA ratios from three biological replicates of WISH cells treated with DMSO or Torin1 (1μM) in combination with IFN β (100pM) for 12 hrs were calculated (Student’s t-test, n = 3) for the genes indicated and are shown as means and 95% confidence intervals. (B) Polysome profiles of WISH cells treated as in A. mRNA abundance was assessed by qPCR for the indicated genes in each fraction. Abs254, absorbance of light at 254nm.

To further confirm that the ISG mRNAs *TLR3*, *NT5C3A(3)* and *RNF19B* are less efficiently translated upon mTOR inhibition, three independent biological replicate isolates of cytoplasmic and polyribosome-associated mRNA from WISH cells treated with IFN β alone or simultaneously with IFN β and Torin1 were assessed by qPCR (Fig 4A). As previous studies have demonstrated that the murine gene *Isg15* encodes an mRNA selectively reliant on the mTOR pathway for its translation [7,11,57], the mTOR dependence of the translation of its human ortholog was also tested. Results confirmed that *TLR3*, *NT5C3A(3)*, and *RNF19B*, but not *ISG15* mRNAs reproducibly exhibit reduced translation efficiency when cells are treated simultaneously with Torin1 and IFN β compared to IFN β alone (Fig 4A). It must be kept in mind, however, that Torin1 treatment results in a significant reduction in *global* (including *ISG15*) mRNA translation, and that *TLR3*, *NT5C3A(3)*, and *RNF19B* are exceptional ISGs in that their mRNAs are *relatively* less translated. To rule out the possibility that some of the mRNAs studied were subject to degradation during isolation of polysome-associated fractions, an additional biological replicate was generated to allow assessment of mRNA levels in six fractions throughout the polysome profile (Fig 4B). This analysis demonstrated that the Torin1-induced reduction in polysome-associated mRNA was accompanied by a corresponding increase in mRNA in lighter fractions (free mRNA and monosomes) for *TLR3*, *NT5C3A(3)*, and *RNF19B*, similar to the positive control *RPL26*. Consistent with previous findings that histone gene translation is resistant to mTOR inhibition [36], *HIST1H1B* mRNA distribution across the polysome profile remained relatively unchanged.

Western blot analyses were performed to assess the relative IFN-induced levels of TLR3 and NT5C3A (no suitable antibodies were found for RNF19B) in the presence or absence of mTOR inhibitors, using the same lysates as in S1A Fig. Again, these lysates were prepared by seeding an equal number of cells, and equal volumes of lysate were loaded to assess total ISG production and maintenance. Notably, basal protein levels and turnover will also impact on the observed protein levels. Rapamycin failed to alter the apparent level of the two proteins, while Torin1 effectively reduced expression of the major IFN-induced immunoreactive TLR3 and NT5C3A bands (S1B Fig). Both TLR3 bands and the lower IFN-inducible NT5C3A band exhibited lower levels even when 2X lysate was loaded to compensate for the approximate 50% reduction in global protein translation (S1B Fig).

### The mTOR-sensitive mRNA NT5C3A(3) contains TOP-like elements

**Fig 5:**
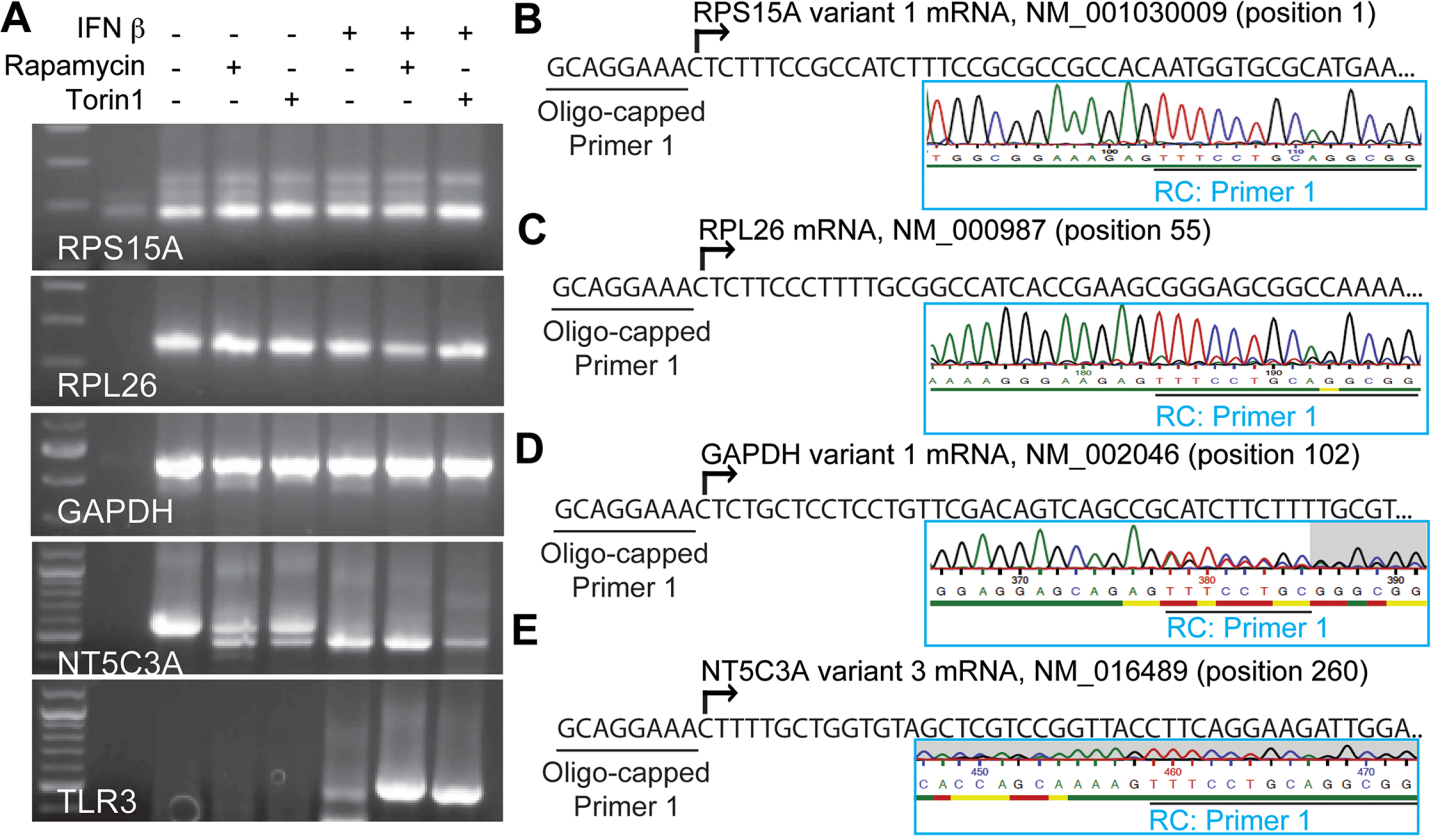
Transcriptional start site determination using an oligo-capping approach. (A) Ethidium bromide stained agarose gels show for each indicated genes the PCR products obtained using an oligo-capped forward primer and nested gene-specific reverse primers to amplify 5’ capped and polyadenylated mRNA from WISH cells treated with DMSO (-), 100nM rapamycin, or 1μM Torin1 alone or in combination with IFN β (100pM) for 12 hrs. (B-E), Major identified transcriptional start sites are shown for each gene as the sequence immediately following that of the oligo-capped primer. Representative Sanger sequencing dendograms (boxes) are shown displaying the reverse complement of the associated sequence. RC, reverse complement.

Real TSSs may not align with those in public databases, may vary from cell type-to-cell type, and are potentially altered in response to stimuli [52,58]. We therefore used an oligo-capping approach to determine the major TSSs for select mRNAs in WISH cells subjected to each of the 6 treatments (control, rapamycin, Torin1, IFN β, rapamycin and IFN β, Torin1 and IFN β). Following oligo-capping and amplification of 5’-capped and 3'-polyadenylated mRNAs and 2 rounds of gene-specific PCR, products were visible on ethidium bromide (EtBr)-stained agarose gels (Fig 5A). Excision and Sanger sequencing of the major bands revealed that the major TSS of *RPS15A* remained unchanged in response to stimulation with IFN and/or mTOR inhibition and represents a TOP sequence (5’-CTCTTTCCG…) in agreement with both dbTSS and NCBI databases (Fig 5B). Similarly, the major TSS for *RPL26* (5’-CTCTTCCCTTTTG…) was shown to be a TOP sequence in agreement with the dbTSS, although the NCBI database indicates this sequence begins at position 55 of the mRNA NM_000987 (Fig 5C). The correct identification of human RPL26 as a true TOP mRNA may be important to explain previous data suggesting that a PRTE distal from an mRNA's 5’-terminus was sufficient to render the mRNA translationally dependent on the mTOR signaling pathway [34]. The major identified TSS for *GAPDH* remained unchanged in response to stimuli, and despite being extremely rich in pyrimidines, the sequence (5’-CTCTGCTCCTCCTG…) fails to adhere to the definition of a TOP-like sequence (Fig 5D). Given the evidence we have presented that *GAPDH* is relatively dependent on mTOR for its translation, this sequence may represent a TOP-like mRNA.

Apparent molecular weights of EtBr-stained oligo-capped PCR products varied from sample-to-sample for *NT5C3A*. This was expected, because the major *NT5C3A* mRNA variant present in cells changes from NM_001002009 to NM_016489 in response to IFN β, and these variants arise from TSSs in exons 1 and 2, respectively (Fig 3D). Sanger sequencing data was performed on all bands and confirmed that the major *NT5C3A* TSS under non-IFN β stimulated conditions was indeed present in exon 1 (data not shown). Similarly, Sanger sequencing confirmed that the major *NT5C3A* TSS under IFN β stimulation conditions was indeed in exon 2, with major TSSs yielding TOP and TOP-like motifs: 5’-CTTTTGCTGGTG, 5’-TCTTTTGCTGGTG and 5’-TTCTTTTGCTGGTG. Of particular note was the appearance of oligo-capped PCR products in response to rapamycin or Torin1 with an apparent molecular weight similar to that of the IFN β-induced *NT5C3A* product (Fig 5A). Sanger sequencing confirmed that this mTOR inhibitor-induced PCR product corresponds to the IFN β-induced *NT5C3A(3)* mRNA (Fig 5E and data not shown) (see Discussion).

Consistent with *TLR3* being an ISG, clear EtBr-stained PCR products were visible in lanes corresponding to IFN-treated samples (Fig 5A). Sanger sequencing results for these PCR products corresponded to the mRNA listed in the NCBI database (NM_003265). The primary TSS identified for TLR3 in these samples, 5’-CTATTTGCCACAC, appears to lack a TOP or TOP-like sequence, as was also the case for the only TSS identified for RNF19B: 5’- TTCTATCGCCGGGA, an mRNA listed in NCBI to have zero 5’UTR.

## Discussion

The findings presented herein appear to contradict previous reports suggesting that type-I IFN signaling hijacks the mTOR pathway to selectively up-regulate the translational efficiency of ISGs. With the exception of NB-4 cells, we could not observe an activation of the mTOR signaling pathway by IFN in five other cell lines analyzed. Recent publications have similarly demonstrated no activation of the mTOR pathway by type-I IFN in multiple cell lines showing rather an induction of autophagy and mTOR inhibition, particularly after sustained treatment with IFN [59,60]. Previously published observations that IFN can activate the mTOR pathway may result from a number of factors, including differences among cell lines, different experimental conditions or use of non appropriate negative control.

The cross-talk model is appealing because it would explain how a cell, even under starvation conditions, is able to mount an anti-viral response when it encounters IFN. Our findings demonstrate that the vast majority of ISG-encoded mRNAs are no more reliant on the mTOR pathway than most other mRNAs. While it is true that mTOR inhibition throughout the course of IFN stimulation can reduce ISG products, this largely results from a global, non-specific, repression of translation efficiency, possibly even related to a reduction in core mRNA translation machinery (*e*.*g*. ribosomal proteins and elongation factors). In line with previous work, our results show that the mRNAs selectively translated in an mTOR-dependent manner include those that contain TOP and TOP-like motifs at their 5’ termini, but also highlight a substantial set of mTOR-sensitive mRNAs that do not appear to harbor such RNA elements. An alternative model to explain how a cell exposed to IFN under starvation conditions can still mount a defense against a virus likely relates to the notion that the infecting virus’ mRNA would also be subjected to greatly suppressed mRNA translation, particularly if the number of ribosomal proteins was reduced. As we and others have previously demonstrated that the ISG product USP18 suppresses IFN signaling as a form of negative feedback [38,61], reduced ISG mRNA translation under reduced nutrient conditions will be accompanied by sustained transcriptional induction of ISGs, resulting in a prolonged IFN response.

The diversity of gene expression and TSS usage from one experimental study to another likely ascribes uniqueness to each microarray gene-expression dataset aiming to identify the mTOR-dependent “translatome”. Previous studies likely failed to identify the mTOR dependence of *NT5C3A* mRNA translation precisely for this reason. That is, in non-IFN stimulated cells, the predominant mRNA transcript has a distinct 5’UTR from the IFN-induced mRNA variant *NT5C3A(3)*, and only this latter contains a functional TOP-like element at its 5’ end. We did observe the striking phenomenon that mTOR inhibitors alone were able to increase the relative abundance of the IFN-induced variant. Although over-interpretation of this non-quantitative PCR result is not advised, an attractive model to explain the ability of mTOR pathway inhibitors to promote the transcription of the IFN-induced *NT5C3A* variant involves the perturbed Akt and/or ERK signaling pathways presented in Fig 2A. It has been shown, for example, that rapamycin activates ATF-1, ATF-2 and CREB transcription factors via ERK and Akt signaling pathways [62]. Notably, the database DECODE (Decipherment of DNA Elements) indicates potential ATF-2 and CRE-BP1 transcription factor binding sites within the promoter of the *NT5C3A* gene in proximity to the STAT1 binding sites.

Why the IFN-induced TLR3 and NT5C3A are subject to mTOR-dependent translational control remains an open question. TLR3 functions as part of the innate immune system to recognize pathogen-associated molecular patterns and double-stranded RNA and activate IRF3. The mTOR pathway has been implicated in the control of proinflammatory cytokine production induced by bacterial stimuli in immune cells (monocytes, macrophages, dendritic cells). Interestingly, in keratinocytes TLR3 stimulation was reported to induce inflammatory cytokines through an mTOR-regulated pathway [63]. Hence, this latter may modulate TLR3 translation itself. NT5C3A is a catabolic enzyme that dephosphorylates pyrimidine nucleoside monophosphates to the corresponding nucleosides [64]. Its activity is most critical during erythroid differentiation when it is upregulated to support the complete breakdown of ribosomes and RNA. Interestingly, NT5C3A, called Lupin in the mouse, was shown to associate with inclusion bodies in lymphocytes from systemic lupus erythematosus and AIDS patients [55,56], and this may be related to the fact that NT5C3A is an ISG.

A recent report has implicated mTORC2 in the ability of IFN to promote the transcription of many common ISGs [65]. As Torin1 inhibits both mTORC1 and mTORC2, one might expect to see a transcriptional reduction in many ISGs in response to Torin1 treatment. In our genome-wide mRNA expression dataset, however we observed an equal number of ISGs with reduced and increased mRNA levels in response to Torin1 treatment during IFN stimulation in our normalized data set. Some outstanding examples of mRNAs strongly induced by IFN but less abundant when cells were also treated with Torin1 include *IFI27*, *BATF2*, *SOCS1*, *APOL3*, and *MUC13*. mRNAs showing the opposite behavior (*i*.*e*. induced by IFN and more strongly induced by IFN and Torin1) include *IDO1*, *PARP14*, *IL6*, *NFE2L3*, and *OPTN*. According to our model, this phenomenon is likely the result of perturbed signaling pathways and the coordinate transcription of mRNA by combinations of transcription factors.

Together our data support a model wherein sustained mTOR inhibitor treatment throughout an IFN stimulation results in reduced translation of TOP, TOP-like and non-TOP mRNAs, and in a repression of global mRNA translation sufficient to partially prevent the appearance of ISG-encoded proteins. Our data also suggest the possibility that, in response to IFN, the activation of transcription factors, including those activated by the Jak/Stat pathway, can alter directly or indirectly the TSS of mRNAs rendering them mTOR-dependent.

## Supporting Information

S1 Fig (PDF)

S1 Table (PDF)

S2 Table (PDF)

S3 Table (PDF)

S4 Table (PDF)
