# Supplementary material for: Assessment of mTOR-Dependent Translational Regulation of Interferon Stimulated Genes
Source: PLoS One. 2015 Jul 24;10(7):e0133482. doi: 10.1371/journal.pone.0133482 (PMC4514843; doi:10.1371/journal.pone.0133482)
Supplement: S1 Fig — (PDF) [file pone.0133482.s001.pdf]

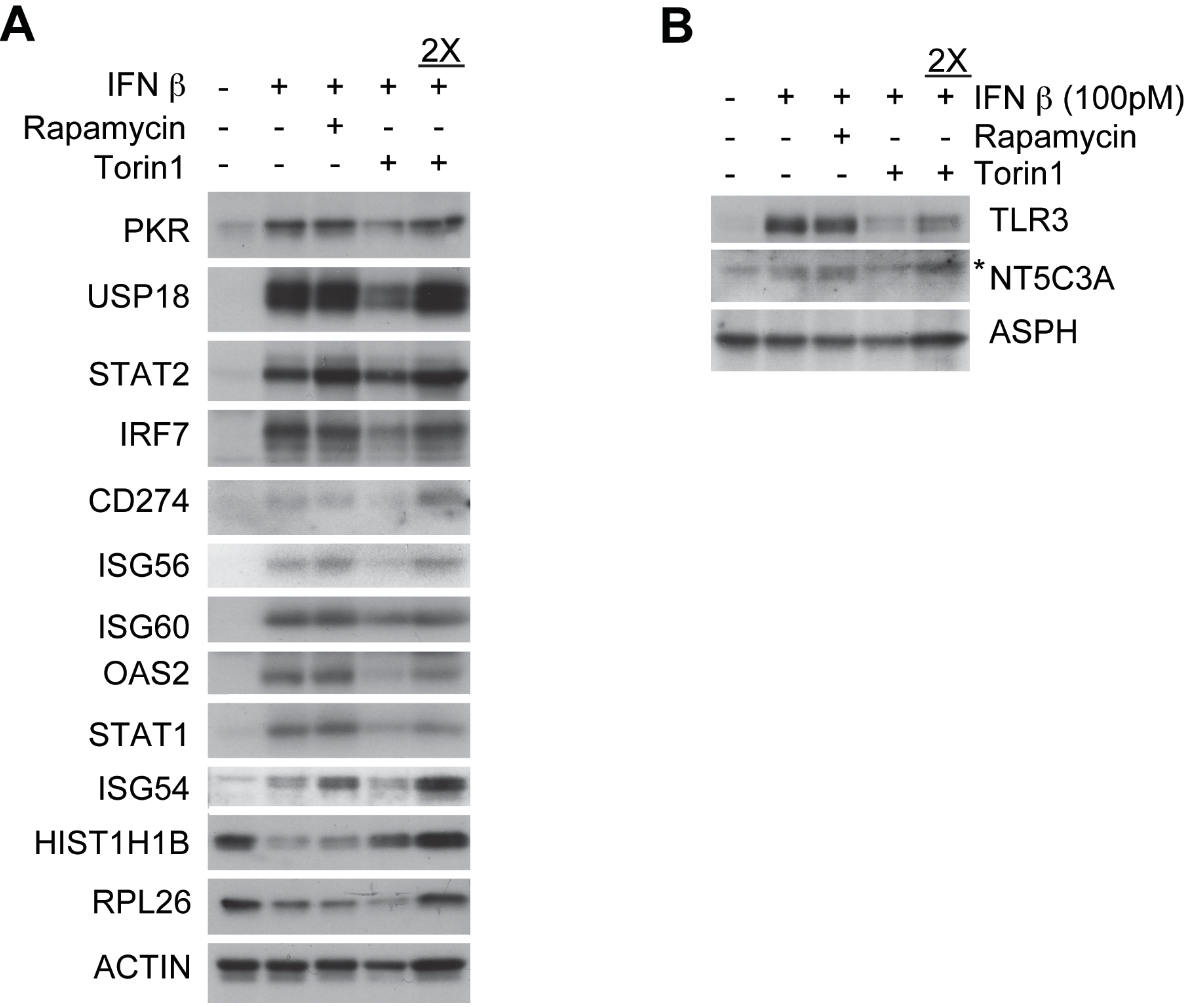

Supplemental Figure 1. Assessment of mTOR inhibitors on IFN-Induced ISG Protein Expression **A)** Western blot analyses comparing expression levels of 11 known ISG-encoded proteins (PKR, USP18, STAT2, IRF7, CD274, ISG56, ISG60, OAS2, STAT1, and ISG54) following treatment of WISH cells with DMSO (-), IFN β (100pM, 12 hrs) alone or in combination with 100nM rapamycin or 1μM Torin1. The IFN-suppressed gene encoded protein HIST1H1B, the TOP-mRNA encoded protein RPL26, and Actin serve as controls. 2X, double the quantity of cellular lysate was loaded/analyzed. **B)** Western blot analyses comparing protein expression levels of TLR3 and NT5C3A following treatment of WISH cells with DMSO (-), IFN β (100pM, 12 hrs) alone or in combination with 100nM rapamycin or 1μM Torin1. 2X, double the quantity of cellular lysate was loaded/analyzed; *, non-interferon induced protein recognized by NT5C3A antibody.

Figure S1.
