# Supplementary material for: Assessment of mTOR-Dependent Translational Regulation of Interferon Stimulated Genes
Source: PLoS One. 2015 Jul 24;10(7):e0133482. doi: 10.1371/journal.pone.0133482 (PMC4514843; doi:10.1371/journal.pone.0133482)
Supplement: S1 Table — (PDF) [file pone.0133482.s002.pdf]

Table S1. Primers used in the study

qPCR (SYBR Green) Primers Forward and Reverse

Gene Name	Forward	Reverse	RefSeq RNAs
ASPH	AGGCGGTGCTGAGGCACA	TGAGAGTCCGCCTTTCCTCCCA	NM_004318.3; NM_032466.3; NM_001164754.1; NM_001164756.1
CD274	TGGTGGTGCCGACTACAAGCG	GGGGTAGCCCTCAGCCTGACAT	NM_014143.3
FTO	ACAGGTCAGCGGTGGCAGTG	CTGCCACTCGGTGGGTGGAAC	NM_001080432.2; NM_001080432.2; NM_001080432.2; NM_001080432.2; NM_001080432.2
GALNT4	TAGGGAGAGAGGAGTTGGGC	TTCCTTTCATGCAGGCGCTA	NM_003774.4
GAPDH	ACAGTCCATGCCATCACTGCC	GCCTGCTTCACCACCTTCTTG	NM_002046.3
IRF7	CCCCATCTTCGACTTCAGAG	AAGGAAGCACTCGATGTCGT	NM_004031.2; NM_001572.3;NM_004029.2
ISG15	GCGAACTCATCTTTGCCAGT	CTTCAGCTCTGACACCGACA	>NM_005101.3
MAGEA3	GATTCTCGCCCTGAGCAACG	CCCACTGGCAGATCTTCTCC	NM_005362.3
MRPS24	CAAGATGGCGGCCTCCGTGTGC	CGTTCGCTCTGCGGCATGGTC	NM_032014.2
NDUFC2	CGCGACGGCTGGTTTGC	AGCAGGTATCAGTGAAACTGGAGCA	NM_004549.5
NT5C3 (1)	CTCTGGGATCCCGCGCTTC	TTCTGGAATTCTGGCATCATCTCG	NM_001002010.2
NT5C3 (2)	GGAAGACGGGGCGGAAGACC	CTGGCATCATTTTCACATGTACGGC	NM_001002009.2
NT5C3 (3)	TGGTGTAGCTCGTCCGGTTACCTT	CTGGCATCATTTTCACATGTACGGC	NM_016489.12
NT5C3 (4)	TGCTGGTGTAGCTCGTCCGGT	TGGCATCTCAGGCAAATCACAGC	NM_001166118.2
RNF19B	GGGCAAGAATCTGGACCAGCAGA	TCAACGTGCCCAGCTGCCAA	NM_153341.2
RPL26	AGGCATTTCAATGCACCTTC	CTGCACCCGTTCAATGTAGA	NM_000987.3
STAT2	TATCACAGCCAGTGCCAGAG	CTGATTCCCATCCTTGGAGA	NM_005419.3; NM_198332.1
TBCD	ACCACGGCCAGCCAGGTGTA	GGCACCACTGTGCGAGGCTT	NM_005993.4
TLR3	CGAGAGTGCCGTCTATTTGCCACA	GGTGGTGGAGGATGCACACAGC	NM_003265.2

mRNA variant-specific qPCR (SYBR Green) Primers Forward and Reverse

Gene	Forward Primer Sequence	Reverse Primer Sequence	RefSeq RNAs
MX1	GGATCCTGAGGCCCGTGTTTGC	GGTGGACTTAGGTGCCTTGTGGC	NM_001144925.1
MX1	TCATTTCTTCGCGGCGCGGG	TGGCCACAGAATTCCAAAGCCCC	NM_002462.3
MX1	GCCACCATTCCAAGCTTACTTTGC	AATGAGGTCGATGCAGGGGCG	NM_001178046.1
OAS1	AACCCAGGCCTGTGATCCTGGACCC	GGCAGGGATGAATGGCAGGGAGG	NM_002534.2
OAS1	TGAGAAGGCAGCTCACGAAACCC	AGTGTGCTGGGTCAGCAGAATCC	NM_001032409.1
OAS2	GAGCCAGTTGCAGAAAACCAGGC	TGGTGTCTGCATTGTCGGCACTTT	NM_016817.2
OAS2	AAACCAGGCCTGTGATCTTGGACC	GGGCTATTTCCAGACAACGCCTCC	NM_002535.2
OAS2	CGGCAGCCAGCTGAGAGCAAT	GAGCCACCTATGGCCACTCCCT	NM_001032731.1
PML	GGCAGAGGAACGCGTTGTGGT	GGCTGGATGACCACGCGGAA	NM_033238.2
PML	GACCCTATTGACGTTGACCTGAGGA	GGTCTGTCTTCTGCTTGGGGGT	NM_033246.2
PML	GGGGAGGCAGGTAGGGAGAGGAA	GCAGGCTGGAGGCTCCTTGTG	NM_033244.3
PML	TTGACGTTGACCTGGATGTCTCCAA	CATCCAGCTCTCGGGAGGACG	NM_033249.2
PML	GAGGCAGAGGAACGCGTTGTGG	TCCTCAGCACAGCTTGGGACTCA	NM_033240.2
PML	TGCCGAAAACTCGTCCTCCCG	TTTCCCGGTTCACCGCAGCC	NM_033238.2
PML	CGTTGACCTGCTGCCTCCTCC	GGGCAAAGAGGCCTGGAATGGT	NM_033247.2
PML	CAGATGCCGAAAACTCGTCCTCC	GGGTAGCCCCAGGAGAACCCAC	NM_002675.3, NM_033249.2, and 033246.2

Sequences of gene-specific Reverse Primers for PCR with PCR Primer 1: 5'-TCATACACATACGATTTAGGTGACACTATAGAGCGGCCGCCTGCAGGAAA-3' for TSS determination

Gene	Reverse Primer Outside	Reverse Primer (Nested)
RPS15Aa	CACCAGAGTCCATGAGGCAT	TGTTTAGCCTGCCTGTGAGG
RPS15Ab	TCCCTCCTGTGTGTTTTCGT	TGGAGCACGGCCTAATAAGC
RPL26a	CATTAGCCTTTTCCCGCTGC	CCTTTCGGATGGGCATGGAT
RPL26b	TTTGGCTTTCCGTTCGAGGA	CATTAGCCTTTTCCCGCTGC
TLR3a	ATTGCGTGAAAACACCCTGG	TGGAGTTCAGTCAAATTCGTGC
TLR3b	TCCAATTGCGTGAAAACACCC	TGAGTTGGACATGAGATGGAGT
RNF19Ba	GCTTGATGTCATCTGCTGGTC	GCAGCATGAACTCCTCGTACT
RNF19Bb	ATGTCATCTGCTGGTCCAGATT	TGAACTCCTCGTACTTGTGCAT
NT5C3a	AGTACATCGCCGATTCCAGC	GCTGAACAAGCAAACCATGTG
NT5C3b	TCCTTTGAGCACCCCAGTTT	AGCATAACGTCAGATTCTGCCA
GAPDHa	AGTGATGGCATGGACTGTGG	GCCTTCTCCATGGTGGTGAA
GAPDHb	TGATGACCCTTTTGGCTCCC	TTCCCGTTCTCAGCCTTGAC

a/b: indicate two different primers for the same gene
