# Supplementary material for: Assessment of mTOR-Dependent Translational Regulation of Interferon Stimulated Genes
Source: PLoS One. 2015 Jul 24;10(7):e0133482. doi: 10.1371/journal.pone.0133482 (PMC4514843; doi:10.1371/journal.pone.0133482)
Supplement: S2 Table — (PDF) [file pone.0133482.s003.pdf]

Table S2. Genes Translationally-Regulated by mTOR Inhibitors

chipID	RefSeq	GeneID	GeneSymbol	GeneDescrip	GO	Rapamycin_t	Rapamycin_t	Rapamycin_p	Rapamycin_p	Rapamycin_c	Rapamycin_c	IFN-beta_cyt	IFN-beta_cytoplasm_FDR
3475_at	NM_001550	3475	'IFRD1	interferon-related develop		-1.2376596	0.00283753	-1.3019019	2.42E-05	-0.6740178	0.04774334	-0.1738128	0.1539398
29117_at	NM_013263	29117	'BRD7	bromodomain containing 7		-0.9152727	0.05472269	-0.8740486	0.02159472	NA	NA	0.01518431	0.9649132
79031_at	NM_024065	79031	'PDCL3	phosducin-like 3		-0.7368492	0.04054804	-0.7362869	0.02965952	NA	NA	-0.0467616	0.84558556
6210_at	NM_0010300	6210	'RPS15A	ribosomal protein S15a		-0.7217874	0.01945377	-0.7529992	0.01117417	NA	NA	-0.0472527	0.87092192
10247_at	NM_005836	10247	'HRSP12	heat-responsive protein 12		-0.6224268	0.00283753	-0.6229554	0.00047363	NA	NA	-0.0332963	0.91543791
28559_at			'			-0.6121391	0.02228114	NA	NA	NA	NA	-0.2848773	0.50753954
653857_at	NM_001164	653857	'ACTR3C	ARP3 actin-related protein		-0.4800066	0.08106837	NA	NA	NA	NA	-0.2428355	0.6798581
28468_at			'			0.42655076	0.06990798	NA	NA	NA	NA	0.21856735	0.35090743
677826_at			'			0.60590229	0.03140084	NA	NA	NA	NA	-0.650456	0.00565354
729974_at	XM_0034034	729974	'LOC729974	ret finger protein-like 4A-li		0.7851676	0.11187615	NA	NA	NA	NA	-0.3571618	0.42135957

Translationally Regulated by Torin1

chipID	RefSeq	GeneID	GeneSymbol	GeneDescrip	GO	Torin1_trans	Torin1_trans	Torin1_polys	Torin1_polys	Torin1_cytop	Torin1_cytop	IFN-beta_cyt	IFN-beta_cytoplasm_FDR
100131205_at			RPL21P28	ribosomal protein L21 pse		-1.4602742	0.00692407	-1.9028848	0.00040215	NA	NA	-0.2966671	0.47372451
6210_at	NM_0010300	6210	'RPS15A	ribosomal protein S15a		-1.4471741	5.61E-05	-1.3573026	0.00035959	NA	NA	-0.0472527	0.87092192
6201_at	NM_001011	6201	'RPS7	ribosomal protein S7		-1.2481386	0.00011778	-1.6205647	1.17E-05	-0.9644187	0.01122682	-0.1928272	0.59281228
729080_at			LOC729080	glycine cleavage system pr		-1.2426215	0.01047563	-1.0318976	0.03738848	NA	NA	-0.2585173	0.69932378
4501_at	NM_005952	4501	'MT1X	metallothionein 1X		-1.1654984	0.0001401	-1.1971697	2.33E-05	NA	NA	-0.0361905	0.86448594
10952_at	NM_006808	10952	'SEC61B	Sec61 beta subunit		-1.092244	9.98E-05	-1.3722922	1.02E-06	-0.6109232	0.00084197	-0.1538783	0.16108274
85236_at	NM_080593	85236	'HIST1H2BK	histone cluster 1, H2bk		-1.0066875	0.00022082	-1.0010158	4.28E-06	NA	NA	0.30876457	0.07140564
387841_at			RPL13AP20	ribosomal protein L13a ps		-1.0057703	0.00813553	-1.7227043	0.00265271	NA	NA	0.10630352	0.86331468
10632_at	NM_006476	10632	'ATP5L	ATP synthase, H+ transpor		-0.9948362	9.32E-06	-0.9493449	9.30E-06	NA	NA	-0.0563928	0.82500925
4833_at	NM_005009	4833	'NME4	non-metastatic cells 4, pro		-0.9729616	0.01178871	-1.1348658	1.40E-05	NA	NA	-0.1427204	0.14225437
6206_at	NM_001016	6206	'RPS12	ribosomal protein S12		-0.949724	0.00038453	-0.9458852	0.00064555	NA	NA	0.15092857	0.46207333
653659_at	NM_0010798	653659	'TMEM183B	transmembrane protein 18		-0.9203958	0.0826329	-1.1178087	0.01797239	NA	NA	0.03731952	0.94484422
23480_at	NM_014302	23480	'SEC61G	Sec61 gamma subunit		-0.9046442	8.69E-06	-0.8850907	1.07E-05	NA	NA	-0.1715201	0.44400176
4729_at	NM_021074	4729	'NDUFV2	NADH dehydrogenase (ubi		-0.8850633	0.00138144	-0.9398186	0.00071862	NA	NA	-0.0694725	0.76748848
9324_at	NM_138730	9324	'HMGN3	high mobility group nuclec		-0.8814743	1.20E-05	-0.8798817	1.21E-05	NA	NA	0.11871378	0.39184755
900_at	NM_199246	900	'CCNG1	cyclin G1		-0.8806392	5.07E-06	-0.8653095	2.38E-06	NA	NA	-0.129551	0.24270757
64951_at	NM_032014	64951	'MRPS24	mitochondrial ribosomal p		-0.8735415	0.00575679	-1.1515809	7.51E-05	NA	NA	-0.2051926	0.24464863
388722_at	NM_0010245	388722	'C1orf53	chromosome 1 open readi		-0.8692329	0.00222277	-1.0638162	8.72E-05	NA	NA	0.02387774	0.95295194
10094_at	NM_005719	10094	'ARPC3	actin related protein 2/3 c		-0.8538396	8.70E-05	-0.9007005	1.50E-05	NA	NA	0.20477934	0.27702743
9200_at	NM_014241	9200	'PTPLA	protein tyrosine phosphat		-0.8518996	5.65E-06	-0.8507153	1.27E-05	NA	NA	-0.2742999	0.0379549
6128_at	NM_0010240	6128	'RPL6	ribosomal protein L6		-0.8475328	5.65E-06	-0.8423483	1.69E-06	NA	NA	-0.1863219	0.1004016
644511_at			RPL13AP6	ribosomal protein L13a ps		-0.8449427	0.0004334	-0.837476	0.00026094	NA	NA	-0.0720357	0.81215577
6191_at	NM_001007	6191	'RPS4X	ribosomal protein S4, X-lin		-0.842963	1.49E-06	-0.862984	1.48E-05	NA	NA	-0.0404302	0.71109172
6207_at	NM_001017	6207	'RPS13	ribosomal protein S13		-0.839687	0.00125739	-0.8671448	0.0004524	NA	NA	0.10414466	0.33324889
6194_at	NM_001010	6194	'RPS6	ribosomal protein S6		-0.8286918	0.00015038	-0.8538945	0.00016569	NA	NA	-0.1505503	0.21364856
6224_at	NM_0011462	6224	'RPS20	ribosomal protein S20		-0.812673	0.00187	-0.7218735	0.00117094	NA	NA	-0.1422452	0.24714048
11224_at	NM_007209	11224	'RPL35	ribosomal protein L35		-0.8125692	6.27E-05	-0.8096379	0.0001171	NA	NA	-0.1217905	0.53640107
8813_at	NM_003859	8813	'DPM1	dolichyl-phosphate manno		-0.8060796	6.47E-05	-0.8551913	9.26E-06	NA	NA	-0.0809311	0.71553295
116150_at	NM_138459	116150	'NUS1	nuclear undecaprenyl pyro		-0.806027	0.01980791	-0.8314877	0.00313126	NA	NA	-0.0984653	0.68183474
11261_at	NM_007236	11261	'CHP	calcium binding protein P2		-0.7999985	1.89E-05	-0.8277942	1.72E-06	NA	NA	-0.0355731	0.748724
1979_at	NM_004096	1979	'EIF4EBP2	eukaryotic translation initi		-0.7997291	1.20E-05	-0.8242348	6.78E-06	NA	NA	-0.2811612	0.11965313
6159_at	NM_000992	6159	'RPL29	ribosomal protein L29		-0.7987206	0.01455698	-0.9030531	0.00280591	NA	NA	-0.0771154	0.8401841
6155_at	NM_000988	6155	'RPL27	ribosomal protein L27		-0.7975442	1.32E-05	-0.7967405	1.84E-05	NA	NA	-0.1590002	0.34717895
6136_at	NM_000976	6136	'RPL12	ribosomal protein L12		-0.7962427	0.00037631	-0.8569464	5.57E-05	NA	NA	-0.2204993	0.26022671
728658_at			RPL13AP5	ribosomal protein L13a ps		-0.7951841	0.00623735	-1.2700538	0.00488444	NA	NA	0.09585709	0.65789947
6176_at	NM_213725	6176	'RPLP1	ribosomal protein, large, P		-0.7888768	0.00050623	-0.9151829	1.63E-05	NA	NA	0.01696713	0.87821792
84833_at	NM_032747	84833	'USMG5	up-regulated during skelet		-0.7826626	0.0023356	-0.7856918	0.00207315	NA	NA	-0.0856855	0.7607409
442907_at			MIR339	microRNA 339		-0.777997	0.06598855	-0.725184	0.01692831	NA	NA	-0.423123	0.22192391
23678_at	NM_170709	23678	'SGK3	serum/glucocorticoid regu		-0.7715509	0.06790888	NA	NA	NA	NA	-0.531132	0.26557194
5935_at	NM_0010174	5935	'RBM3	RNA binding motif (RNP1,		-0.7715028	2.97E-05	-0.8504893	3.04E-05	NA	NA	-0.0420863	0.77281147
6154_at	NM_000987	6154	'RPL26	ribosomal protein L26		-0.7668673	0.00074182	-0.8085836	0.00064966	NA	NA	-0.1275025	0.75680756
10983_at	NM_006835	10983	'CCNI	cyclin I		-0.7582324	1.20E-05	-0.7582409	1.50E-05	NA	NA	0.11929099	0.46155942
5204_at	NM_145896	5204	'PFDN5	prefoldin subunit 5		-0.7305387	0.00022585	-0.7462244	0.00057236	NA	NA	-0.1065722	0.71520675
9443_at	NM_004270	9443	'MED7	mediator complex subunit		-0.7273019	4.93E-05	-0.7274621	6.20E-05	NA	NA	0.28854031	0.48888924
3242_at	NM_002150	3242	'HPD	4-hydroxyphenylpyruvate		-0.7237396	0.00085548	-0.6860522	5.73E-05	NA	NA	-0.3306594	0.00397319
6227_at	NM_001024	6227	'RPS21	ribosomal protein S21		-0.7225319	0.00191616	-0.7098815	0.00201121	NA	NA	-0.0564093	0.7526292
131076_at	NM_0010179	131076	'CCDC58	coiled-coil domain contain		-0.7172515	0.00015768	-0.7348118	2.07E-05	NA	NA	-0.1876232	0.57288455
3178_at	NM_031157	3178	'HNRNPA1	heterogeneous nuclear rib		-0.7166245	0.00011778	-0.7190189	6.83E-05	NA	NA	-0.0507114	0.82793372
29937_at	NM_013349	29937	'NENF	neudesin neurotrophic fac		-0.7145415	0.0015031	-0.5890726	0.0007474	NA	NA	0.00322395	0.97667681
10381_at	NM_006086	10381	'TUBB3	tubulin, beta 3 class III		-0.7139146	0.00915279	-1.0528973	4.39E-05	NA	NA	0.15102457	0.44196872
348926_at			FAM86EP	family with sequence simil		-0.710776	0.05176886	-0.8488911	0.00152642	NA	NA	-0.2339	0.29080594
4666_at	NM_005594	4666	'NACA	nascent polypeptide-assoc		-0.7086937	0.00043904	-0.7110189	6.99E-05	NA	NA	0.01125637	0.94782562
6181_at	NM_001004	6181	'RPLP2	ribosomal protein, large, P		-0.6942562	9.32E-06	-0.7014941	9.49E-06	NA	NA	0.08299057	0.43275346
60481_at	NM_021814	60481	'ELOVL5	ELOVL fatty acid elongase		-0.6921362	2.22E-05	-0.7067266	7.06E-06	NA	NA	-0.0932491	0.52650476
10491_at	NM_006371	10491	'CRTAP	cartilage associated protei		-0.6895859	2.13E-06	-0.6822607	6.38E-06	NA	NA	-0.0673196	0.49456328
386677_at	NM_198691	386677	'KRTAP10-1	keratin associated protein		-0.686571	0.05969182	NA	NA	NA	NA	-0.3343805	0.43292228
7098_at	NM_003265	7098	'TLR3	toll-like receptor 3		-0.6859212	0.00029692	-0.6907271	0.00023531	NA	NA	3.4970398	7.69E-09
6189_at	NM_001006	6189	'RPS3A	ribosomal protein S3A		-0.6845894	0.08402031	NA	NA	NA	NA	-0.0938772	0.89101454
84154_at	NM_032194	84154	'RPF2	ribosome production facto		-0.6773396	0.02160972	-0.65767	0.00677693	NA	NA	-0.6998257	0.01628393
1033_at	NM_005192	1033	'CDKN3	cyclin-dependent kinase in		-0.676608	0.00080463	-0.6787413	5.94E-05	NA	NA	-0.3251002	0.19337634
6130_at	NM_000972	6130	'RPL7A	ribosomal protein L7a		-0.6727847	0.00221468	NA	NA	NA	NA	-0.123523	0.38673531
26986_at	NM_002568	26986	'PABPC1	poly(A) binding protein, cy		-0.6691052	0.01337722	NA	NA	0.77169704	0.00030732	-0.3100957	0.13771267
10750_at	NM_006613	10750	'GRAP	GRB2-related adaptor prot		-0.6688362	0.00662278	-0.7323841	0.01180042	NA	NA	-0.1687946	0.67610632
6156_at	NM_000989	6156	'RPL30	ribosomal protein L30		-0.6668006	2.14E-05	-0.6610333	2.48E-05	NA	NA	0.01634789	0.91667661
254863_at	NM_152766	254863	'C17orf61	chromosome 17 open reac		-0.6636555	0.00024612	-0.6628178	0.00016979	NA	NA	-0.0527722	0.78709555
9768_at	NM_014736	9768	'KIAA0101	KIAA0101		-0.6569615	2.51E-05	-0.662366	1.70E-05	NA	NA	-0.0098998	0.94288247
5501_at	NM_002710	5501	'PPP1CC	protein phosphatase 1, cat		-0.6539091	1.49E-05	-0.6421891	1.46E-05	NA	NA	-0.1701586	0.12027589
8637_at	NM_003732	8637	'EIF4EBP3	eukaryotic translation initi		-0.6525657	0.08755758	-0.6439697	0.04735852	NA	NA	-0.090397	0.86877502
8667_at	NM_003756	8667	'EIF3H	eukaryotic translation initi		-0.6453342	1.46E-05	-0.6747137	7.89E-06	NA	NA	-0.0199358	0.90167645
6203_at	NM_001013	6203	'RPS9	ribosomal protein S9		-0.6385287	0.00767884	NA	NA	NA	NA	-0.178747	0.22231255
4736_at	NM_007104	4736	'RPL10A	ribosomal protein L10a		-0.6354738	8.62E-05	NA	NA	NA	NA	-0.2990511	0.05585957
3646_at	NM_001568	3646	'EIF3E	eukaryotic translation initi		-0.6229964	0.00225123	-0.668105	9.79E-05	NA	NA	-0.2868579	0.27599092
25898_at	NM_015436	25898	'RCHY1	ring finger and CHY zinc fir		-0.6169434	0.00062557	-0.6098945	0.00035488	NA	NA	-0.1928181	0.33046154
58505_at	NM_021227	58505	'OSTC	oligosaccharyltransferase c		-0.6112777	7.19E-05	-0.6040785	6.40E-05	NA	NA	-0.2374336	0.07568076
81853_at	NM_030969	81853	'TMEM14B	transmembrane protein 14		-0.6079462	0.00444028	-0.6016986	0.00347752	NA	NA	-0.2702601	0.07700989
79412_at	NM_145348	79412	'KREMEN2	kringle containing transme		-0.6071334	0.02616791	-0.5912206	0.01533592	NA	NA	-0.2901705	0.35974041
4673_at	NM_139207	4673	'NAP1L1	nucleosome assembly pro		-0.6048033	0.00140097	-0.6033196	0.00036504	NA	NA	-0.1026747	0.49978067

29880_at	NM_013338	29880	'ALG5	asparagine-linked glycosyl	-0.601527	0.00064207	-0.6016181	4.54E-05	NA	NA	-0.1926426	0.16166209
1936_at	NM_001130(	1936	'EEF1D	eukaryotic translation elor	-0.5933191	0.00040295	-0.662594	6.12E-05	NA	NA	-0.0715292	0.48563981
100652919_a	XM_0034035	100652919	'LOC1006529	uncharacterized LOC10065	-0.5897814	0.11752177	NA	NA	NA	NA	-0.026818	0.92655892
51389_at	NM_016104	51389	'RWDD1	RWD domain containing 1	-0.5851404	0.00054631	-0.5851337	5.94E-05	NA	NA	0.11011822	0.31310975
10412_at	NM_014886	10412	'NSA2	NSA2 ribosome biogenesis	-0.5823666	0.00092489	NA	NA	NA	NA	-0.1200554	0.68463649
26225_at	NM_177985	26225	'ARL5A	ADP-ribosylation factor-lik	-0.5767232	0.01531964	NA	NA	NA	NA	0.06650727	0.75741977
892_at	NM_005190	892	'CCNC	cyclin C	-0.5737241	0.00013547	NA	NA	NA	NA	0.0932522	0.56028178
8665_at	NM_003754	8665	'EIF3F	eukaryotic translation initi	-0.572427	1.53E-05	NA	NA	NA	NA	-0.1266136	0.19478718
6135_at	NM_001199	6135	'RPL11	ribosomal protein L11	-0.5716596	9.95E-05	-0.6164678	3.35E-05	NA	NA	0.00562189	0.96085465
6152_at	NM_000986	6152	'RPL24	ribosomal protein L24	-0.5673277	0.01452899	NA	NA	NA	NA	-0.1016599	0.46094598
54982_at	NM_017882	54982	'CLN6	ceroid-lipofuscinosis, neur	-0.5624712	0.00126492	NA	NA	NA	NA	-0.2840874	0.03764064
7178_at	NM_003295	7178	'TPT1	tumor protein, translation	-0.555955	0.00011871	NA	NA	NA	NA	0.03716614	0.72786614
1938_at	NM_001961	1938	'EEF2	eukaryotic translation elor	-0.5494743	0.00015038	NA	NA	NA	NA	-0.1073704	0.29414378
3615_at	NM_000884	3615	'IMPDH2	IMP (inosine 5'-monophos	-0.5475732	0.00099269	-0.6017476	4.27E-05	NA	NA	-0.3396706	0.00698448
51451_at	NM_016309	51451	'LCMT1	leucine carboxyl methyltra	-0.5345975	0.00019331	NA	NA	NA	NA	0.194317	0.06812583
7507_at	NM_000380	7507	'XPA	xeroderma pigmentosum,	-0.5308315	0.00165354	NA	NA	NA	NA	0.05997776	0.60862577
6158_at	NM_001136	6158	'RPL28	ribosomal protein L28	-0.529512	0.00019489	NA	NA	NA	NA	-0.0184788	0.8875043
9349_at	NM_000978	9349	'RPL23	ribosomal protein L23	-0.5220463	0.00025624	NA	NA	NA	NA	-0.6801599	0.00066467
10597_at			TRAPPC2P1	trafficking protein particle	-0.5139287	0.01180816	NA	NA	NA	NA	0.27908851	0.06516437
9412_at	NM_004264	9412	'MED21	mediator complex subunit	-0.503435	0.00010747	NA	NA	NA	NA	0.07135604	0.86076713
3945_at	NM_002300	3945	'LDHB	lactate dehydrogenase B	-0.5009864	2.46E-05	NA	NA	NA	NA	-0.1162746	0.22707476
341_at	NM_001645	341	'APOC1	apolipoprotein C-I	-0.4953062	0.00506024	NA	NA	NA	NA	0.03312913	0.85843381
57099_at	NM_020371	57099	'AVEN	apoptosis, caspase activati	-0.4873977	0.0002123	NA	NA	NA	NA	0.23037373	0.03355653
9045_at	NM_003973	9045	'RPL14	ribosomal protein L14	-0.4870217	0.00014454	NA	NA	NA	NA	-0.0079628	0.95968327
3476_at	NM_001551	3476	'IGBP1	immunoglobulin (CD79A) l	-0.4793713	0.06778978	NA	NA	0.70536355	0.00032827	-0.1684998	0.26071817
51018_at	NM_016052	51018	'RRP15	ribosomal RNA processing	-0.4693202	0.012083	NA	NA	NA	NA	0.008289	0.970003
10286_at	NM_005872	10286	'BCAS2	breast carcinoma amplifie	-0.4681486	0.000753	NA	NA	NA	NA	0.16360984	0.42063685
10480_at	NM_006360	10480	'EIF3M	eukaryotic translation initi	-0.4659528	1.58E-05	NA	NA	NA	NA	-0.0390856	0.83351801
6230_at	NM_001028	6230	'RPS25	ribosomal protein S25	-0.4574032	0.07152005	-0.5852604	0.01174404	NA	NA	0.0225269	0.89052065
8723_at	NM_003794	8723	'SNX4	sorting nexin 4	-0.4473157	0.00901711	NA	NA	NA	NA	0.05019322	0.7884586
115106_at	NM_138443	115106	'HAUS1	HAUS augmin-like complex	-0.4456078	0.00573989	NA	NA	NA	NA	0.01219669	0.97168614
100124536_at			SNORA38B	small nucleolar RNA, H/AC	-0.4306193	0.004123	NA	NA	NA	NA	-0.3282752	0.24557329
23478_at	NM_014300	23478	'SEC11A	SEC11 homolog A (S. cerev	-0.4271173	0.0158033	NA	NA	NA	NA	-0.1654364	0.16600776
6902_at	NM_004607	6902	'TBCA	tubulin folding cofactor A	-0.4213102	0.11777384	NA	NA	NA	NA	-0.0191444	0.96346229
10910_at	NM_006704	10910	'SUGT1	SGT1, suppressor of G2 all	-0.4203181	0.00788803	NA	NA	NA	NA	-0.0566147	0.65018274
6138_at	NM_002948	6138	'RPL15	ribosomal protein L15	-0.4193597	0.10690118	NA	NA	0.59008009	0.0032163	-0.6569598	0.00023157
4048_at	NM_001256	4048	'LTA4H	leukotriene A4 hydrolase	-0.4159542	0.02438355	NA	NA	NA	NA	-0.3576265	0.03344578
6139_at	NM_001199	6139	'RPL17	ribosomal protein L17	-0.4124894	0.07815256	NA	NA	NA	NA	-0.6574079	0.0050801
2764_at	NM_004124	2764	'GMFB	glia maturation factor, bet	-0.4118994	0.00341443	NA	NA	NA	NA	-0.2611084	0.50773789
10069_at	NM_016940	10069	'RWDD2B	RWD domain containing 2	-0.390636	0.01276223	NA	NA	NA	NA	0.11218174	0.32278032
51251_at	NM_001002(	51251	'NT5C3	5'-nucleotidase, cytosolic I	-0.3848481	0.01415928	NA	NA	NA	NA	2.54038977	1.54E-05
10527_at	NM_006391	10527	'IPO7	importin 7	-0.3786162	0.00104814	NA	NA	NA	NA	-0.0415181	0.76460276
10651_at	NM_001006(	10651	'MTX2	metaxin 2	-0.3756515	0.06888504	NA	NA	NA	NA	-0.1228829	0.59334797
55643_at	NM_017797	55643	'BTBD2	BTB (POZ) domain contain	-0.3698878	0.03687782	NA	NA	NA	NA	-0.3272866	0.02135843
6124_at	NM_000968	6124	'RPL4	ribosomal protein L4	-0.3695017	0.00481227	NA	NA	NA	NA	-0.2050781	0.06770672
51290_at	NM_016570	51290	'ERGIC2	ERGIC and golgi 2	-0.3636532	0.00575679	NA	NA	NA	NA	-0.209615	0.4254109
566_at	NM_001700	566	'AZU1	azurocidin 1	-0.3589728	0.10572029	NA	NA	NA	NA	-0.2964674	0.14845757
8649_at	NM_021970	8649	'LAMTOR3	late endosomal/lysosomal	-0.3462422	0.00420543	NA	NA	NA	NA	-0.2158279	0.26071817
116254_at	NM_138785	116254	'C6orf72	chromosome 6 open readi	-0.3395523	0.08987933	NA	NA	NA	NA	0.25106079	0.15833696
80306_at	NM_025205	80306	'MED28	mediator complex subunit	-0.3273055	0.02450879	NA	NA	NA	NA	0.05692154	0.74349784
8634_at	NM_003729	8634	'RTCD1	RNA terminal phosphate c	-0.3261021	0.08739151	NA	NA	NA	NA	0.11514178	0.53908608
26796_at			SNORD53	small nucleolar RNA, C/D b	-0.3226576	0.14415657	NA	NA	NA	NA	-0.1984807	0.48801136
138241_at	NM_182505	138241	'C9orf85	chromosome 9 open readi	-0.3200587	0.0504993	NA	NA	NA	NA	0.176885	0.24676144
6125_at	NM_000969	6125	'RPL5	ribosomal protein L5	-0.3115795	0.0150684	NA	NA	NA	NA	-0.232259	0.0668966
51187_at	NM_016304	51187	'RSL24D1	ribosomal L24 domain con	-0.3022508	0.04343795	NA	NA	NA	NA	-0.4381217	0.22174449
137886_at	NM_001077(	137886	'UBXN2B	UBX domain protein 2B	-0.2956892	0.14908705	NA	NA	NA	NA	-0.2122455	0.21438349
112942_at	NM_080667	112942	'CCDC104	coiled-coil domain contain	-0.2649145	0.11285314	NA	NA	NA	NA	-0.170157	0.41043607
7423_at	NM_003377	7423	'VEGFB	vascular endothelial growt	-0.2642631	0.09013529	NA	NA	NA	NA	-0.1544868	0.26532201
127544_at	NM_153341	127544	'RNF19B	ring finger protein 19B	-0.2503402	0.13553389	NA	NA	NA	NA	1.6868988	1.28E-07
6845_at	NM_005638	6845	'VAMP7	vesicle-associated membra	-0.2350769	0.08180948	NA	NA	NA	NA	-0.1933244	0.11977041
83939_at	NM_032025	83939	'EIF2A	eukaryotic translation initi	-0.1709316	0.14092942	NA	NA	0.62516923	0.02374934	0.07586441	0.71289411
9318_at	NM_004236	9318	'COPS2	COP9 constitutive photom	-0.1051817	0.12522705	NA	NA	NA	NA	-0.1727091	0.52006181
5266_at	NM_002638	5266	'PI3	peptidase inhibitor 3, skin	0.29325974	0.14186788	NA	NA	NA	NA	0.28745637	0.13644795
100132386_a	NM_001146(	100132386	'KRTAP4-9	keratin associated protein	0.37789044	0.13252563	NA	NA	NA	NA	0.07939249	0.77987135
9677_at	NM_001024	9677	'PPIP5K1	diphosphoinositol pentaki	0.37883436	0.01090472	NA	NA	NA	NA	-0.0783184	0.61476573
23600_at	NM_001167	23600	'AMACR	alpha-methylacyl-CoA race	0.40204255	0.03792882	NA	NA	NA	NA	0.065843	0.60679812
677826_at			'		0.48452784	0.10244229	NA	NA	NA	NA	-0.650456	0.00565354
1469_at	NM_001898	1469	'CST1	cystatin SN	0.5516616	0.00075864	NA	NA	NA	NA	-0.1371939	0.43539639
26818_at			'		0.5547199	0.00276255	NA	NA	NA	NA	0.18703267	0.591891
26832_at			'		0.5940555	0.04597662	NA	NA	NA	NA	-0.4378916	0.14984778
118429_at	NM_058172	118429	'ANTXR2	anthrax toxin receptor 2	0.67540387	0.00061994	0.88709308	0.00017553	NA	NA	-0.0157742	0.9384673
150737_at	NM_152517	150737	'TTC30B	tetratricopeptide repeat d	0.72361907	0.00307623	NA	NA	NA	NA	0.04949488	0.74048132
79633_at	NM_024582	79633	'FAT4	FAT tumor suppressor hon	0.90041962	0.00984864	1.21718498	3.60E-05	0.59132584	0.00494088	0.11406759	0.48522843
1778_at	NM_001376	1778	'DYNC1H1	dynein, cytoplasmic 1, hea	1.10310241	0.00023164	1.45854477	1.26E-06	0.82814735	0.00104459	-0.130924	0.48814893
154_at	NM_000024	154	'ADRB2	adrenergic, beta-2-, recept	1.11104709	1.20E-05	1.05146304	8.22E-06	NA	NA	0.53843094	0.00276818
6080_at			'		1.26401409	0.00040748	1.26754839	0.00037936	NA	NA	-0.1186663	0.62500308
3097_at	NM_006734	3097	'HIVEP2	human immunodeficiency	1.3270975	0.00479548	1.97220651	5.34E-07	1.23329849	0.0011217	0.41248685	0.07607099
114907_at	NM_148177	114907	'FBXO32	F-box protein 32	2.08702661	0.001129	2.15051661	4.24E-07	1.41578042	1.83E-05	-0.1822645	0.14336927
