# Supplementary material for: Assessment of mTOR-Dependent Translational Regulation of Interferon Stimulated Genes
Source: PLoS One. 2015 Jul 24;10(7):e0133482. doi: 10.1371/journal.pone.0133482 (PMC4514843; doi:10.1371/journal.pone.0133482)
Supplement: S3 Table — (PDF) [file pone.0133482.s004.pdf]

Table S3. TOP and TOP-like Sequences in mRNAs less efficiently translated in response to Torin1 treatment during an IFN stimulation

GENE	TOP (NCBI)	TOP (dbTSS)	TOP-like (dbTSS)
EEF2	ctcttccg	ctcttccg	
EIF2A	ctctttccg	ctctttccg	
EIF3H	ctctttcttcctg	ctctttcttcctg	
PABPC1	cccttctccccg	cccttctccccg	
RPL14	cttctcg	cttctcg	
RPL15	cctttccg	cctttccg	
RPL28	ctctttccg	ctctttccg	
RPL30	cctttctcg	cctttctcg	
RPS12	ctctttccctg	ctctttccctg	
RPS15A	ctctttccg	ctctttccg	
RPS9	ctctttctcag	ctctttctcag	
RPL35	cttcctctttccctcg	ctctttccctcg	
RPL4	cttttcctg	ccttttcctg	
RPLP1	cctttcctcag	ccctttcctcag	
RPS25	cttcctttttg	ctttttg	
RPS6	cctcttttccg	ctcttttccg	
TPT1	cccccccg	cttttccg	
EEF1D		ccctttca	
EIF3E		cttttctttg	
EIF3F		cttctttctcg	
EIF3M		cttttccg	
IPO7		ccttttcg	
NAP1L1		cttttttta	
NSA2		ctttcctg	
RPL11		ctcttcctg	
RPL23		ctttttttctttttttccg	
RPL24		cttttcg	
RPL26		ctcttcccttttg	
RPL29		ctcttccg	
RPL5		ccttttccca	
RPL7A		ctttctctctcctcccg	
RPLP2		cttttcctccctg	
RPS20		ctttttg	
RPS21		ctttctctctcg	
RPS3A		ccctttttg	
TLR3			actttcg
CCDC104			ctctcg
COPS2			aatttctcctccccctcccg
GINM1			acctcccg
GMFB			attctta
NT5C3A			cttttg
RPL10A			tctcttttccg
RPL12			gctttcg
RPL17			tctttcccta
RPS13			ctttcg
RPS7			tctcttccta
RSL24D1			ctctca
RTCD1			cttccg
RWDD1			ctcccg
RWDD2B			tttttttttccg
