# Supplementary material for: Assessment of mTOR-Dependent Translational Regulation of Interferon Stimulated Genes
Source: PLoS One. 2015 Jul 24;10(7):e0133482. doi: 10.1371/journal.pone.0133482 (PMC4514843; doi:10.1371/journal.pone.0133482)
Supplement: S4 Table — (PDF) [file pone.0133482.s005.pdf]

Table S4. Interferon-stimulated Genes and Primers

chipID	RefSeq	GeneID	GeneSymbol	IFN-beta_cytoplasm_log2ratio	IFN-beta_cytoplasm_FDR	Ensembl (Interferome	Type 1 (Interferome)	Type 2 (Ir	Type 3 (In	Forward Primer Sequence (qPCR c	Reverse Primer Sequence (qPCR C	Isoforms Detected (qPCR Confirmation)	Interferon-Induced (1=qPCR Confirmation)
10964_at	NM_006820	10964	IFI44L	6.743565663	8.74E-12	ENSG00000137959	8	3	2	TGTTGGCAAAAGTGAAGCAA	GGGTCCAGTTCCAAATCTGA	NM_006820.2	1
4600_at	NM_002463	4600	MX2	6.37498225	2.97E-11	ENSG00000183486	12	3	2	TGATTTCTCCATCCTGAACGTG	CATGTGCTGTCTCCCTGTCA	NM_002463.1	1
54739_at	NM_199139	54739	XAF1	6.130022759	0	ENSG00000132530	0	1	0	GACTTCTCGGTGTGCAGGAA	CCTGGCACTCATTGGCCTTA	NM_017523.3	1
3429_at	NM_001130080	3429	IFI27	6.088855308	1.69E-10	ENSG00000165949	13	3	3	CGGTGAGGTCAGCTTCACAT	TGAAGCCCATGGCACTGAG	NM_001130080.1; NM_005532.3	1
4939_at	NM_016817	4939	OAS2	6.069110857	5.83E-12	ENSG00000111335	13	4	3	AAACCAGGCCTGTGATCTTGGACC	GGGCTATTTCCAGACAACGCCTCC	NM_002535.2	1
4599_at	NM_002462	4599	MX1	5.96993356	5.83E-12	ENSG00000157601	15	4	2	GTGCATTGCAGAAGGTCAGA	TTCAGGAGCCAGCTGTAGGT	NM_001144925.1, NM_002462.3, NM_001178046.1	1
219285_at	NM_152703	219285	SAMD9L	5.262005868	4.99E-10	ENSG00000177409	1	0	1	ACAGCAAAAGTTCAAATGGCCT	CTTCTGGCAAGGGGCTTACA	NM_152703.2	1
23586_at	NM_014314	23586	DDX58	5.118185895	4.99E-10	ENSG00000107201	10	4	3	GCATGACCACCGAGCAGCGA	AGCCACGGAACCAGCCTTCCT	NM_014314.3	1
3965_at	NM_009587	3965	LGALS9	5.108129845	4.68E-07	ENSG00000168961	6	4	0	GCCCGAGGAGAGGAAGACA	CCTGGGTGGGAAACAGACAG	NM_009587.2	1
8743_at	NM_003810	8743	TNFSF10	5.090527542	5.49E-10	ENSG00000121858	0	1	0	GCTTCAGTGACCGGCTGCCT	ATTTTCTGCAAACTGCTTCAGCTCG	NM_001190943.1	1
3434_at	NM_001548	3434	IFIT1	4.932364741	6.77E-07	ENSG00000185745	17	3	2	TCTCAGAGGAGCCTGGCTAA	TCAGGCATTTCATCGTCATC	NM_001548.3	1
2633_at	NM_002053	2633	GBP1	4.862967569	4.81E-09	ENSG00000117228	15	6	2	ACGACAGGGTCCAGTTGCTGA	TGCCTTTCGTCGTCTCATTTTCGT	NM_002053.2	1
10346_at	NM_006074	10346	TRIM22	4.700718777	4.80E-09	ENSG00000132274	12	4	2	GGTTGAGGGGATCGTCAGTA	AGAACTTGCAGCATCCCACT	NM_006074.4; NM_001199573.1	1
3433_at	NM_001547	3433	IFIT2	4.560659674	2.50E-10	ENSG00000119922	15	6	2	CAGCTGCCTGAACCGAGCCC	GCATTCCAGGGCTGCCTCGT		1
116071_at	NM_138456	116071	BATF2	4.53744584	3.21E-08					TGAAGAAGCAGAAGAACCGGG	TGAGCAGGAGGCACAATCCA	NM_138456.3	1
64135_at	NM_022168	64135	IFIH1	4.470357833	4.99E-10	ENSG00000115267	8	2	3	AGAAGGAGGTCTGGGGCATGGA	CTCCGGGGATGCTCTTGCTGC	NM_022168.2	1
3620_at	NM_002164	3620	IDO1	4.33090325	9.83E-10	ENSG00000131203	5	2	0				
3437_at	NM_001549	3437	IFIT3	4.096779793	7.67E-10	ENSG00000119917	15	4	3	TTTTCGGAACAGCAGAGACA	AATGGCATTTCAGCTGTGG	NM_001549.4	1
83666_at	NM_031458	83666	PARP9	4.027982236	5.09E-09					GTTCCTGGGTGCTGACCGT	AGAGCGAGGTAATCCTACCTGA	NM_031458.2	1
10561_at	NM_006417	10561	IFI44	4.017770912	1.98E-08	ENSG00000137965	11	5	2	TCCAAGGGCATGTAACGCAT	CCTTCAGCGATGGGGAATCA	NM_006417.4	1
51191_at	NM_016323	51191	HERC5	3.897028314	9.09E-10	ENSG00000138646	6	1	2				
8638_at	NM_198213	8638	OASL	3.796447059	2.22E-10	ENSG00000135114	11	5	2	CGCCTGGTGAAACACTGGTA	GGTGAAGCCTTCGTCCAACA	NM_003733.3	1
2537_at	NM_022873	2537	IFI6	3.679616166	5.77E-09	ENSG00000126709	19	5	2	CGACGGCCATGAAGGTCAGG	CATGCGGCAGAAGGCGGTAT	;NM_022873.2; NM_022872.2; NM_002038.3	1
55008_at	NM_017912	55008	HERC6	3.610677236	4.39E-09	ENSG00000138642	2	0	1	ACCTGATACCTGCAGAAACCC	GGCGACTTCGTCTGACTCTAA	NM_017912.3	1
9881_at	NM_014831	9881	TRANK1	3.5902201	2.69E-08	ENSG00000168016	0	1	0	ACACGGGCTCGGGTCAACCT	CCAGCACTGGTGCTTGGCGT	NM_014831.2	
27074_at	NM_014398	27074	LAMP3	3.582751123	9.94E-09	ENSG00000078081	5	3	1	CCTGCAGGTGAAAACAACCG	ATGGCCCCAATCACAGGAAG	NM_014398.3	1
91351_at	NM_001012967	91351	DDX60L	3.545574629	4.39E-09					CTCCGAAGCTCTTTGGACCT	CATCGTTGCTGCTTCTTGGG	NM_001012967.1	1
9636_at	NM_005101	9636	ISG15	3.538743734	7.67E-10	ENSG00000187608	20	5	3	GCGAACTCATCTTTGCCAGT	CTTCAGCTCTGACACCGACA	NM_005101.3	1
9246_at	NM_198183	9246	UBE2L6	3.514756741	2.44E-09	ENSG00000156587	10	2	3	ACACTCGGTCCCGACATGAT	CATGGGAGGCTTGAACGGAT	NM_004223.4	1
7098_at	NM_003265	7098	TLR3	3.497039804	7.69E-09	ENSG00000164342	6	3	0	CGAGAGTGCCGTCTATTTGCCACA	GGTGGTGGAGGATGCACACAGC	NM_003265.2	1
54809_at	NM_017654	54809	SAMD9	3.451431291	2.69E-05	ENSG00000205413	3	1	2	ATCTCATGGTTGCAGTGGGT	GGTCCATGTGTGATGCCCATA	NM_017654.3	1
2635_at	NM_018284	2635	GBP3	3.376414171	4.39E-09	ENSG00000117226	4	3	0	GGAGTCTGTGACCGATGCAA	TCTCCTTTAGTACTCGGGCCT	NM_018284.2	1
11274_at	NM_017414	11274	USP18	3.347631181	9.94E-09	ENSG00000184979	0	1	0	ACTCCTTGATTTGCGTTGAC	TTTCCCACGGGTCTTCTT	NM_017414.3	1
23424_at	NM_014290	23424	TDRD7	3.30658578	1.04E-08	ENSG00000196116	7	2	2	CAGTGCAAAGGCATGTGACC	GAGGCAGGAGGCTTGACATT	NM_014290.2	1
84941_at	NM_032855	84941	HSH2D	3.291336288	3.07E-08	ENSG00000196684	1	0	1	CGCAAACGTGGATTACGAGG	TCATCTCTGCTGACGGCTTC	NM_032855.2	1
3665_at	NM_004030	3665	IRF7	3.264669061	1.55E-08	ENSG00000185507	15	4	1	CCCCATCTTCGACTTCAGAG	AAGGAAGCACTCGATGTCGT	;NM_004031.2; NM_001572.3;NM_004029.2	1
54979_at	NM_017878	54979	HRASLS2	3.245671977	4.56E-08					CTCGCTTTGGCTATGCACAC	AATAAGGCAACTCCTGCCCC	NM_017878.1	1
79132_at	NM_024119	79132	DHX58	3.227468488	2.12E-09	ENSG00000108771	0	1	0	TGCTCATCCATGACACCGTC	ATACCCCGAGGGCTGTTAGA	NM_024119.2	1
3669_at	NM_002201	3669	ISG20	3.216868791	1.06E-08	ENSG00000172183	15	5	3	AGAGTGGCCTGGCTCGTTGC	GACCCACCACCAGCTTGCCT	NM_002201.4	1
24138_at	NM_012420	24138	IFIT5	3.21193555	3.65E-09	ENSG00000152778	11	3	3	ACACCTTGAAGGCCATTCTG	CAAGCTGTTGCCCAATTGTA	NM_012420.2	1
3431_at	NM_080424	3431	SP110	3.191696874	5.77E-09	ENSG00000135899	0	1	0	GCAGCCTCAGGACCAGCTGAAA	CGCACAAACCATGCCACCGT	NM_080424.2	1
6737_at	NM_003141	6737	TRIM21	3.161178354	5.27E-09	ENSG00000132109	8	3	1	TGCCACAGCTCAGCACTGGAAC	TTCAGCCCTGGCACATGGCAC	NM_003141.3	1
54625_at	NM_017554	54625	PARP14	3.119336315	5.08E-09					AAAGCCACCCTGCCAGATAC	AGGCCCTCTACTAAGCACGA	NM_017554.2	1
10379_at	NM_006084	10379	IRF9	3.1117736	3.98E-09	ENSG00000213928	11	3	3	CGCTGCACCCGAAAACTCCG	TGCGGCCCCTCTCAGGAACC	NM_006084.4	1
55601_at	NM_017631	55601	DDX60	3.089378839	4.51E-09	ENSG00000137628	0	1	0	CCCAGGGTCCAGGATTTTAT	GAACAGTTGCTGCCACTTGA	NM_017631.5	1
80830_at	NM_030641	80830	APOL6	3.087368214	9.26E-09	ENSG00000196785	4	1	3	ACCAGGCGGAGAGAGAAAGT	CGTCTTGTAGCTCCACGTCTT	NM_030641.3	1
91543_at	NM_080657	91543	RSAD2	3.086057554	1.61E-07	ENSG00000134321	10	4	2	GGTGGAGAGCCATTTCTTCA	TCCCTACACCACCTCCTCAG	NM_080657.4	1
6773_at	NM_198332	6773	STAT2	3.070857276	4.99E-10	ENSG00000170581	9	3	1	TATCACAGCCAGTGCCAGAG	CTGATTCCCATCCTTGGAGA	NM_005419.3; NM_198332.1	1
6398_at	NM_003004	6398	SECTM1	3.060338049	2.00E-09	ENSG00000141574	2	2	0	CAGGAGAGCGCCATCTTCAA	GTACCAGGCGAACATGACCA	NM_003004.2	1
85441_at	NM_033405	85441	PRIC285	3.030414324	1.00E-07	ENSG00000130589	0	1	0	ACAGATGGTGGCCTTCGACC	GTGACACGCACTCCATCAAG	NM_001037335.2	1
151636_at	NM_138287	151636	DTX3L	3.002979293	1.51E-07	ENSG00000163840	1	0	0	TTCAGTGAAAGGGCAGCTAAG	GTATGCCCTCTGCTCTTTGGA	NM_138287.3	1
55281_at	NM_018295	55281	TMEM140	2.975270017	2.39E-09	ENSG00000146859	2	0	2	ATCTGCCTGCCATTTCCCTTC	ATCTCTACTGCCCTCTGCCG	NM_018295.3	1
51296_at	NM_016582	51296	SLC15A3	2.95461894	4.81E-09	ENSG00000110446	1	1	0	GGTGGTGGCGTTTATTCAGC	CTGGAAGTTGGCGATGTCCT	NM_016582.2	1
7318_at	NM_003335	7318	UBA7	2.884601122	2.24E-07	ENSG00000182179	5	1	0	AGATTATCCCAGCCATTGCCA	GCCCAGCTGGTACCTTCAGA	NM_003335.2	1
25939_at	NM_015474	25939	SAMHD1	2.881759885	4.29E-09	ENSG00000101347	4	2	1	CGAGGTTCTTGACTGCTGTG	GCAGTAATGCGCCTGTGATT	NM_015474.3	1
9473_at	NM_004848	9473	THEMIS2	2.856268591	5.03E-07	ENSG00000130775	2	1	1	AATGAGTGCTGCCTCTCCAC	TGTGACAATCCTGTGGGTCG	NM_001105556.1; NM_004848.2	1
5359_at	NM_021105	5359	PLSCR1	2.844385968	9.94E-09	ENSG00000188313	15	3	3	TCCCCGAGCGCCAGC	TCCGGGTGAGAAGCATTCAT	NM_021105.2	
85363_at	NM_033093	85363	TRIM5	2.816935148	6.06E-08	ENSG00000132256	3	1	1	GCATCGGCTGCAGGGGTCAG	GGAGCCACTGTCACATCAACCCAG	NM_033034.2	1
8542_at	NM_145344	8542	APOL1	2.756231678	1.53E-08	ENSG00000100342	2	1	0	AAGATTCCTTGGAGGAGGCCC	GTTTTGTTGCACCCTCGCTC	NM_003661.3	1
55337_at	NM_018381	55337	C19orf66	2.753502933	4.99E-10	ENSG00000130813	0	1	0	GCAGTGCGGACCCTCG	TGTCCCGGTCCTGCTTCATA	NM_018381.2	1
373856_at	XM_036729	373856	USP41	2.745065781	3.10E-06	ENSG00000161133	1	1	0	GGATTATAGACACGGCCTGGTT	GCAGCAGAAGCATCTGGAAAG	XM_036729.9	1
6672_at	NM_003113	6672	SP100	2.714324854	8.22E-07	ENSG00000067066	0	1	0	AGGTGTGCAACAAATGGGGA	CGGTTCTGAGGCGAAAAAGC	NM_001080391.1	1
4940_at	NM_006187	4940	OAS3	2.684443945	3.66E-10	ENSG00000111331	8	2	2	CGCCAGGTTGCGGCTCAGAA	TGGGATCAGCGGGGTCCAGG	NM_006187.2	1
23037_at	NM_015022	23037	PDZD2	2.66270865	1.55E-08	ENSG00000133401	1	0	0	ATGCTCCTCTGACCACAAGC	TGATCCTCGGCCTCCACTAA	NM_178140.2	1
629_at	NM_001710	629	CFB	2.653966154	1.92E-08	ENSG00000166285	5	3	0	TTCCGGGAAAGTGATGTGGG	GTGTGTCCTGGCTTGCTTTG	NM_001710.5	1
5920_at	NM_004585	5920	RARRES3	2.590494205	4.50E-08	ENSG00000133321	4	2	1	CGTGATCCATCTGGCTCCTC	CACCTGTTTACAGCGGGACT	NM_004585.3	1
6772_at	NM_139266	6772	STAT1	2.582165335	4.57E-09	ENSG00000115415	18	2	3	TTGGCAGTTTTCTTCTGTCA	CACGCTCTCGCTCCTT	NM_007315.3, NM_139266.2	1
162394_at	NM_144975	162394	SLFN5	2.554529903	1.79E-07	ENSG00000166750	1	0	1	AGGTTCTCTGCTCTGGACTTG	CTTGATTATGCCCCCACCAGA	NM_144975.3	1
3569_at	NM_000600	3569	IL6	2.548027372	8.69E-07	ENSG00000136244	5	1	1	AATGAGGAGACTTGCCTGGTG	TGGGTCAGGGGTGGTTATTG	NM_000600.3	1
51251_at	NM_001002009	51251	NT5C3	2.540389772	1.54E-05	ENSG00000122643	2	0	1	TGGTGTAGCTCGTCCGGTTACCTT	CTGGCATCATTTTCACATGTACGGC	NM_016489.12	1
710_at	NM_001032295	710	SERPING1	2.50960477	3.88E-09	ENSG00000149131	5	2	0	CAGGAACTCACACCAGCGAT	GAGCTGGAGCTGGTAGCATT	NM_000062.2	1

5698_at	NM_148954	5698	PSMB9	2.505671821	3.48E-09	ENSG00000204261	12	3	3	GGGTTCTGATTCCCGAGTGT	CCTGACCTCCTTCACGTTGG	NM_002800.4	1
4938_at	NM_016816	4938	OAS1	2.449063721	2.00E-09	ENSG00000089127	16	5	3	TGAGAAGGCAGCTCACGAAACCC	AGTGTGCTGGGTCAGCAGAATCC	NM_001032409.1	1
5371_at	NM_033250	5371	PML	2.431306133	2.20E-08	ENSG00000140464	9	4	2	GGGGAGGCAGGTAGGGAGAGGAA	GCAGGCTGGAGGCTCCTTGTG	NM_033244.3	1
3430_at	NM_005533	3430	IFI35	2.423607375	3.03E-09	ENSG00000068079	14	3	2	ACCCATGTCAGCCCCACTGGA	GAGCAGAGCCCGCAAGCAGAG	NM_005533.4	1
10475_at	NM_006355	10475	TRIM38	2.416192346	1.81E-07	ENSG00000112343	4	2	1	AGAAGCTGTCCACAGCAATGCGA	GCTCTTCAGCCCCAGACCAGC	NM_006355.2	1
3075_at	NM_001014975	3075	CFH	2.332546614	2.03E-07	ENSG00000000971	1	1	0	AGGATATGTAACAGCAGATGGTGA/	GAACCAGTGGTGCTTCCAGTA	NM_000186.3	1
27071_at	NM_014395	27071	DAPP1	2.329608368	4.01E-08	ENSG00000070190	1	0	1	CCGGGCTGTACTCTCTCTCT	CTGTGGGCACAAGGTCATCT	NM_014395.2	1
80380_at	NM_025239	80380	PDCD1LG2	2.318616125	3.92E-07					CAGTGTTCTGCGCCTAAAGC	GGTCCTGGGTTCCATCTGAC	NM_025239.3	1
2766_at	NM_006877	2766	GMPR	2.30497347	1.11E-06	ENSG00000137198	4	3	0	TGTTTGAGAGGAACGGACGG	GGGTCACCCGGATGAATGTT	NM_006877.3	1
3659_at	NM_002198	3659	IRF1	2.296480663	5.27E-09	ENSG00000125347	12	6	1	CGACCGCCGAATCGCTCCTG	TCGGCCTGTGTGAATGGCCC	NM_002198.2	1
9603_at	NM_004289	9603	NFE2L3	2.272745335	3.12E-05	ENSG00000050344	2	0	1				
115361_at	NM_052941	115361	GBP4	2.215644271	2.58E-08	ENSG00000162654	3	1	0	GTCCGTTTCTCGAGGATCCAG	ACCACGGGCTGAGAAATCTT	NM_052941.4	1
834_at	NM_033295	834	CASP1	2.205474767	4.43E-06	ENSG00000137752	11	4	1	TCGCTTTCTGCTCTTCCACA	CACTTCCTGCCCACAGACAT	NM_033292.3; NM_001223.4; NM_033293.3	1
5610_at	NM_002759	5610	EIF2AK2	2.162261446	1.40E-07	ENSG00000055332	15	3	3	TCTACGCTTTGGGGCTAA	GCCATCCCGTAGGTCTGT	;NM_002759.3; NM_001135651.2; NM_001135652.2	1
8651_at	NM_003745	8651	SOCS1	2.124820589	7.34E-06	ENSG00000185338	5	3	1	CACTTCCGCACATTCCGTTC	AGGCCATCTTCACGCTAAGG	NM_003745.1	1
64761_at	NM_022750	64761	PARP12	2.123640607	3.56E-09	ENSG00000059378	0	1	0	GCCATTTTTGTGGACGCCAT	ACACACTGTTCACGCAGCTA	NM_022750.2	1
57169_at	NM_021035	57169	ZNFX1	2.123220812	1.53E-07	ENSG00000124201	0	1	0	GCCTTGCAGTTTGCTCTCAC	GACTCGTTGGTTAGGAGGGC	NM_021035.2	1
5157_at	NM_006207	5157	PDGFRL	2.062416234	6.84E-08	ENSG00000104213	4	1	0	GCTACCCTGCGTATCTGGAC	ATTCACCTGTGTCTGCCGAG	NM_006207.2	1
51056_at	NM_015907	51056	LAP3	2.051947096	7.04E-09	ENSG00000002549	2	0	1	AAGCCGGGGGATGTTGTTAG	TCGAAGAGAGGCATCCTCCA	NM_015907.2	1
968_at	NM_001251	968	CD68	2.010165966	5.09E-09					CAGCCTAGCTGGACTTTGGG	AGTGCTCTCTGTAACCGTGG	NM_001251.2	1
87178_at	NM_033109	87178	PNPT1	2.005775533	2.23E-07	ENSG00000138035	3	1	1	CCTGTTGGGGCAGTACGAAT	TCACAATCTCTGGCGAAGGG	NM_033109.3	1
54847_at	NM_017699	54847	SIDT1	1.996218238	2.69E-07								
11119_at	NM_194441	11119	BTN3A1	1.968599456	8.60E-07	ENSG00000026950	1	1	0				
80833_at	NM_145639	80833	APOL3	1.95204831	1.41E-08	ENSG00000128284	5	3	1				
129607_at	NM_207315	129607	CMPK2	1.908035516	5.54E-07	ENSG00000134326	0	1	0				
58191_at	NM_022059	58191	CXCL16	1.901316436	8.17E-07	ENSG00000161921	1	0	0				
3133_at	NM_005516	3133	HLA-E	1.900780996	6.90E-09	ENSG00000204592	0	1	0				
10133_at	NM_021980	10133	OPTN	1.898335679	7.38E-08	ENSG00000123240	1	0	0				
84166_at	NM_032206	84166	NLRC5	1.882189372	4.46E-07	ENSG00000140853	0	1	0				
221756_at	NM_182544	221756	MGC39372	1.880685052	8.72E-07								
4133_at	NM_031846	4133	MAP2	1.852561753	1.18E-05								
4907_at	NM_002526	4907	NT5E	1.838031511	8.22E-06								
80021_at	NM_024956	80021	TMEM62	1.830454192	1.49E-06								
715_at	NM_001733	715	C1R	1.774589853	2.58E-08								
159091_at	NM_138819	159091	FAM122C	1.761677262	1.38E-07								
9111_at	NM_004688	9111	NMI	1.757834744	5.21E-07	ENSG00000123609	8	1	1				
51131_at	NM_001040444	51131	PHF11	1.746874331	8.60E-07	ENSG00000136147	2	0	2				
634_at	NM_001712	634	CEACAM1	1.737489773	6.29E-08	ENSG00000079385	1	0	0				
10384_at	NM_197974	10384	BTN3A3	1.73422711	3.16E-06	ENSG00000111801	1	1	0				
5696_at	NM_148919	5696	PSMB8	1.692169017	2.58E-07	ENSG00000204264	7	2	0				
132321_at	NM_173487	132321	C4orf33	1.688908945	9.72E-05								
127544_at	NM_153341	127544	RNF19B	1.686898795	1.28E-07	ENSG00000116514	1	0	0	GGGCAAGAATCTGGACCAGCAGA	TCAACGTGCCCAGCTGCCAA	NM_153341.2	1
56667_at	NM_033049	56667	MUC13	1.685490157	5.95E-06								
56829_at	NM_024625	56829	ZC3HAV1	1.682152092	8.07E-08	ENSG00000105939	4	0	2				
9830_at	NM_033221	9830	TRIM14	1.672722529	6.72E-08	ENSG00000106785	5	2	1				
837_at	NM_033307	837	CASP4	1.671093446	9.42E-05	ENSG00000196954	3	2	0				
30844_at	NM_139265	30844	EHD4	1.667868211	2.21E-07	ENSG00000103966	1	0	1				
1435_at	NM_172211	1435	CSF1	1.661487725	1.25E-07	ENSG00000184371	1	1	0				
64108_at	NM_022147	64108	RTP4	1.652248546	1.14E-07	ENSG00000136514	4	2	1	GGCAAAACCCCGGGCCACAT	GGCGGAAGCCCAACTTCGCT	NM_022147.2	1
6890_at	NM_000593	6890	TAP1	1.643824149	1.41E-07	ENSG00000206233	9	2	2	GGCTCAGCCGATACCTTCACTCG	GGCCTCGCACCAGGTACCAC	NM_000593.5	1
3428_at	NM_005531	3428	IFI16	1.639294876	5.84E-08	ENSG00000163565	9	4	1	AGCTGAGAGCCATCCCCACA	GCTGGTCATGAACGGTCCTGGAA	NM_001206567.1	1
4615_at	NM_002468	4615	MYD88	1.6362192	4.27E-08	ENSG00000172936	6	1	1				
3673_at	NM_002203	3673	ITGA2	1.632830345	4.40E-05	ENSG00000164171	1	1	0				
93349_at	NM_138402	93349	SP140L	1.631741055	0.000131536								
3660_at	NM_002199	3660	IRF2	1.63099015	1.47E-06	ENSG00000168310	7	3	1	ATGCAGAAAGCGAAACGACT	CTGTTGTAAGGCACCGGATT	NM_002199.3	1
3134_at	NM_018950	3134	HLA-F	1.621130082	1.43E-07	ENSG00000137403	0	1	0				
11118_at	NM_007047	11118	BTN3A2	1.616429981	1.78E-06	ENSG00000172062	0	1	0				
134429_at	NM_139164	134429	STARD4	1.603941255	1.43E-07								
10516_at	NM_006329	10516	FBLN5	1.578505034	4.39E-07								
3627_at	NM_001565	3627	CXCL10	1.567951102	9.91E-06	ENSG00000169245	10	4	0	AACCTCCAGTCTCAGCACCA	ACACGTGGACAAAATTGGCT		1
51513_at	NM_016135	51513	ETV7	1.557473142	2.58E-07	ENSG00000010030	1	0	0				
9683_at	NM_153029	9683	N4BP1	1.547142927	1.06E-05	ENSG00000102921	2	0	1				
58985_at	NM_021258	58985	IL22RA1	1.546136657	2.00E-06								
6495_at	NM_005982	6495	SIX1	1.520745759	5.70E-05	ENSG00000126778	1	0	0				
26010_at	NM_015535	26010	SPATS2L	1.513444622	1.33E-06	ENSG00000196141	0	1	0				
23780_at	NM_145637	23780	APOL2	1.508480501	5.55E-08	ENSG00000128335	2	1	1				
2120_at	NM_001987	2120	ETV6	1.502267212	4.51E-07	ENSG00000139083	2	0	1				
3106_at	NM_005514	3106	HLA-B	1.498228089	3.77E-07	ENSG00000204523	4	2	0				
441108_at	NM_001207003	441108	C5orf56	1.477426259	0.000231572								
840_at	NM_033340	840	CASP7	1.473000319	6.87E-07	ENSG00000165806	5	4	1	AGTGACAGGTATGGGCGTTC	CGGCATTTGTATGGTCCTCT	NM_033338.4; NM_033339.3; NM_001227.3; NM_03	1
64167_at	NM_022350	64167	ERAP2	1.467425162	0.000152468	ENSG00000213689							
3964_at	NM_201545	3964	LGALS8	1.459635017	2.75E-07	ENSG00000038210							
8519_at	NM_003641	8519	IFITM1	1.452412813	1.43E-07	ENSG00000178685	14	2	3	CAAAGCCAGAAGATGCACAA	ATGAGGATGCCCAGAATCAG	NM_003641.3	1
197259_at	NM_152649	197259	MLKL	1.44874745	4.18E-06								
51752_at	NM_016442	51752	ERAP1	1.447779587	1.00E-05								

11054_at	NM_007346	11054	OGFR	1.446509175	2.83E-06	ENSG00000060491	2	1	1				
6891_at	NM_018833	6891	TAP2	1.428602102	3.92E-07	ENSG00000206235	4	1	1				
11277_at	NM_033628	11277	TREX1	1.425738029	3.23E-07	ENSG00000213689	4	2	1				
55300_at	NM_018323	55300	PI4K2B	1.42386231	2.56E-06	ENSG00000038210	1	0	1				
84875_at	NM_032789	84875	PARP10	1.403445768	1.90E-06	ENSG00000178685	0	1	0				
25780_at	NM_170672	25780	RASGRP3	1.38663025	8.60E-07								
7052_at	NM_198951	7052	TGM2	1.384369113	6.40E-07								
5699_at	NM_002801	5699	PSMB10	1.356175226	1.99E-05	ENSG00000205220	0	1	0				
55080_at	NM_018009	55080	TAPBPL	1.348460544	2.04E-06								
51667_at	NM_016118	51667	NUB1	1.338815017	1.23E-05								
24145_at	NM_015368	24145	PANX1	1.333419725	3.18E-05								
684_at	NM_004335	684	BST2	1.326065454	1.18E-05	ENSG00000130303	13	4	3				
323_at	NM_173075	323	APBB2	1.323750843	9.91E-07	ENSG00000163697	1	1	0				
58472_at	NM_021199	58472	SQRDL	1.322063019	1.95E-06								
51703_at	NM_203380	51703	ACSL5	1.307008507	0.000199578								
25801_at	NM_012198	25801	GCA	1.306180454	0.000120786	ENSG00000115271	2	0	1				
63901_at	NM_198847	63901	FAM111A	1.304698975	5.48E-06								
23070_at	NM_015050	23070	FTSJD2	1.300345061	2.11E-07	ENSG00000137200	3	0	0				
56243_at	NM_019590	56243	KIAA1217	1.29783796	2.13E-05	ENSG00000120549	1	0	1				
355_at	NM_152877	355	FAS	1.286970569	1.94E-05	ENSG00000026103	2	2	0	ATAAGCCCTGTCCTCCAGGT	TGGAAGAAAAATGGGCTTTG	NM_152871.2, NM_152872.2, NM_000043.4	1
7453_at	NM_213646	7453	WARS	1.284184535	1.47E-06	ENSG00000140105	8	1	0				
29126_at	NM_014143	29126	CD274	1.276844852	9.67E-06	ENSG00000120217	0	1	0	TGGTGGTGCCGACTACAAGCG	GGGGTAGCCCTCAGCCTGACAT	NM_014143.3	1
25865_at	NM_016457	25865	PRKD2	1.268432652	6.87E-05	ENSG00000105287	2	1	1				
7091_at	NM_007005	7091	TLE4	1.266731291	1.61E-05	ENSG00000106829	1	1	0				
340061_at	NM_198282	340061	TMEM173	1.256169319	9.49E-07								
79845_at	NM_024787	79845	RNF122	1.2492302	2.54E-05								
152926_at	NM_152542	152926	PPM1K	1.242696797	6.76E-05	ENSG00000163644	1	0	0				
7706_at	NM_005082	7706	TRIM25	1.241268599	2.24E-07	ENSG00000121060	5	2	1	AGACCTGCTCTCCCGCGTCC	GCTCTCGACGCCCCGTTGAT	NM_005082.4	1
26157_at	NM_015660	26157	GIMAP2	1.233205887	4.00E-06								
81894_at	NM_031212	81894	SLC25A28	1.230011096	6.17E-06								
84333_at	NM_032373	84333	PCGF5	1.226662688	0.000625191	ENSG00000180628	1	0	0				
4343_at	NM_020963	4343	MOV10	1.219838408	6.87E-07	ENSG00000155363	0	1	0				
84248_at	NM_032288	84248	FYTTD1	1.21850101	7.06E-05								
54464_at	NM_019001	54464	XRN1	1.212652051	1.84E-05	ENSG00000114127	1	0	1				
1890_at	NM_001953	1890	TYMP	1.211419122	0.001024931	ENSG00000025708	1	0	0				
8548_at	NM_003666	8548	BLZF1	1.209494048	5.94E-05	ENSG00000117475	1	0	0				
81844_at	NM_030961	81844	TRIM56	1.199934842	7.64E-07	ENSG00000169871	2	1	1	CCTCAGGCCCCCTCACCATCC	GCGAGGACCCGTGGGAAACC	NM_030961.1	1
153684_at				1.193581439	1.44E-05								
79668_at	NM_024615	79668	PARP8	1.191469045	7.64E-05								
59345_at	NM_021629	59345	GNB4	1.191056137	3.22E-05								
4992_at	NM_012360	4992	OR1F1	1.189313221	0.003207712								
57674_at	NM_020914	57674	RNF213	1.180973331	0.000100078	ENSG00000173821	0	1	0				
2323_at	NM_001459	2323	FLT3LG	1.180528672	0.000364091								
685_at	NM_001729	685	BTC	1.166238503	6.61E-06								
2737_at	NM_000168	2737	GLI3	1.165220035	0.000947151								
113277_at	NM_145041	113277	TMEM106A	1.162219868	1.52E-06	ENSG00000184988	0	1	0				
1997_at	NM_172373	1997	ELF1	1.161867392	9.72E-06	ENSG00000120690	1	1	0				
1832_at	NM_004415	1832	DSP	1.160888215	2.22E-05	ENSG00000096696	1	1	0				
255324_at	NM_001013442	255324	EPGN	1.141758383	0.007066403								
80765_at	NM_181900	80765	STARD5	1.140579621	3.60E-05								
2332_at	NM_002024	2332	FMR1	1.14019136	5.99E-05	ENSG00000102081	1	1	0	CACCTCAAAGCGAGCACATA	CAATAGCAGTGACCCCAGGT	NM_002024.5; NM_001185075.1; NM_001185082.1;	1
28996_at	NM_022740	28996	HIPK2	1.126367473	0.0006156								
1316_at	NM_001008490	1316	KLF6	1.118881309	6.88E-05								
81622_at	NM_030930	81622	UNC93B1	1.112360234	8.99E-06	ENSG00000110057	1	0	1				
716_at	NM_201442	716	C1S	1.099401544	2.10E-05	ENSG00000182326	4	2	0				
8676_at	NM_003764	8676	STX11	1.094649919	0.001508331								
10068_at	NM_173043	10068	IL18BP	1.089248141	5.99E-05								
222255_at	NM_152749	222255	ATXN7L1	1.085098054	6.88E-05								
3107_at	NM_002117	3107	HLA-C	1.084820023	1.18E-05	ENSG00000204525	0	1	0				
51474_at	NM_016357	51474	LIMA1	1.081599389	3.72E-06								
8850_at	NM_003884	8850	KAT2B	1.081165983	0.000152468								
8737_at	NM_003804	8737	RIPK1	1.077656292	5.59E-05	ENSG00000137275	1	0	1				
1847_at	NM_004419	1847	DUSP5	1.073103479	0.000158628	ENSG00000138166	1	0	1				
3136_at				1.071034061	1.23E-05								
440689_at	NM_001161334	440689	HIST2H2BF	1.070296719	0.015184075								
54458_at	NM_018457	54458	PRR13	1.067490116	0.01094246								
65979_at	NM_001048183	65979	PHACTR4	1.064111163	0.00018001								
1267_at	NM_033133	1267	CNP	1.06356794	9.91E-07								
103_at	NM_015841	103	ADAR	1.061785729	2.34E-06	ENSG00000160710	6	3	1	GGGCCACTTCCAGTGCGGAG	TGCTTCTGGGAGCTGCCCCT	NM_001111.4 ; NM_015840.3; NM_015841.3;	1
9582_at	NM_004900	9582	APOBEC3B	1.05678085	0.000357935	ENSG00000179750	1	1	0				
23503_at	NM_015346	23503	ZFYVE26	1.053135873	5.95E-06								
100507025_at				1.049681979	0.00336734								
55802_at	NM_018403	55802	DCP1A	1.045390231	1.80E-05								
1545_at	NM_000104	1545	CYP1B1	1.038419312	0.000151396								
3398_at	NM_002166	3398	ID2	1.034943502	6.16E-05								

84930_at	NM_032844	84930	MASTL	1.03450537	9.26E-06								
57664_at	NM_020904	57664	PLEKHA4	1.030410255	0.000343995	ENSG00000105559	2	1	0				
659_at	NM_033346	659	BMPR2	1.030100856	0.000402381								
950_at	NM_005506	950	SCARB2	1.028377685	5.09E-05								
10903_at	NM_181873	10903	MTMR11	1.027127003	9.56E-06	ENSG00000014914	1	1	0				
164022_at	NM_178230	164022	PPIAL4A	1.025602994	0.015808935								
10673_at	NM_006573	10673	TNFSF13B	1.013935649	1.46E-05	ENSG00000102524	3	1	0				
10866_at	NM_006674	10866	HCP5	1.004875913	9.90E-06	ENSG00000206337	1	0	0				
3554_at	NM_000877	3554	IL1R1	1.000823748	7.07E-05								
6303_at	NM_002970	6303	SAT1	1.000397261	0.000538228	ENSG00000130066	1	1	0				
4208_at	NM_002397	4208	MEF2C	0.993931584	1.18E-05								
253260_at	NM_152756	253260	RICTOR	0.981634887	1.74E-05								
55014_at	NM_017919	55014	STX17	0.978389277	0.000182945								
91461_at	NM_138370	91461	PKDCC	0.97785696	2.22E-06								
6238_at	NM_004587	6238	RRBP1	0.977504811	0.003815703								
55450_at	NM_018584	55450	CAMK2N1	0.971850057	0.000231572								
961_at	NM_001025080	961	CD47	0.967080111	0.000109991	ENSG00000196776	3	1	1				
160897_at	NM_180989	160897	GPR180	0.966715376	0.000116412								
144402_at	NM_153634	144402	CPNE8	0.963349491	0.001457401								
11011_at	NM_006852	11011	TLK2	0.958732067	0.038985784								
26270_at	NM_018438	26270	FBXO6	0.9521241	0.000106462								
161145_at	NM_182526	161145	TMEM229B	0.952102546	0.002715753								
1992_at	NM_030666	1992	SERPINB1	0.951873129	0.000188494	ENSG00000021355	1	1	0				
339122_at	NM_198490	339122	RAB43	0.949917278	0.006829518								
51427_at	NM_016220	51427	ZNF107	0.945451286	0.000376706	ENSG00000196247	0	1	0				
114769_at	NM_052889	114769	CARD16	0.945040754	0.002335159								
7726_at	NM_003449	7726	TRIM26	0.943197453	2.56E-05	ENSG00000137313	2	1	0	GCTCGGGACCGGTTGCTTCA	TCCAGGCTCCGTAGTGGGGC	NM_001242783.1	1
4824_at	NM_006167	4824	NKX3-1	0.941348402	0.000332623								
4061_at	NM_002346	4061	LY6E	0.940299248	5.21E-06	ENSG00000160932	6	2	1				
4683_at	NM_001024688	4683	NBN	0.939574846	0.0047657								
4012_at	NM_175920	4012	LNPEP	0.938003904	3.85E-06								
10544_at	NM_006404	10544	PROCR	0.935282376	7.93E-05	ENSG00000101000	1	1	0				
57475_at	NM_020715	57475	PLEKHH1	0.931684357	0.000253938								
4929_at	NM_173173	4929	NR4A2	0.931352653	0.002992982								
79056_at	NM_024081	79056	PRRG4	0.930830214	0.000384837								
8829_at	NM_003873	8829	NRP1	0.929468999	0.001306984								
2069_at	NM_001432	2069	EREG	0.928294707	2.95E-05								
9267_at	NM_017456	9267	CYTH1	0.927288096	6.76E-05	ENSG00000108669	1	0	0				
9674_at	NM_014656	9674	KIAA0040	0.926401938	0.000116412								
6507_at	NM_004172	6507	SLC1A3	0.924031629	0.000102875								
6993_at	NM_006519	6993	DYNLT1	0.923267392	0.008628948	ENSG00000146425	1	1	0				
51479_at	NM_020740	51479	ANKFY1	0.919031315	7.97E-06	ENSG00000185722	1	0	1				
4322_at	NM_002427	4322	MMP13	0.917976347	0.000219026								
5329_at	NM_002659	5329	PLAUR	0.917897201	9.26E-06	ENSG00000121351	1	0	0				
8436_at	NM_004657	8436	SDPR	0.915822875	0.000372549								
7128_at	NM_006290	7128	TNFAIP3	0.913713611	0.000288809								
23325_at	NM_015275	23325	KIAA1033	0.910422499	0.00126932								
89927_at	NM_033201	89927	C16orf45	0.906860318	5.48E-05								
55603_at	NM_017633	55603	FAM46A	0.906836746	0.001297935	ENSG00000112773	0	1	0				
10678_at	NM_033252	10678	B3GNT2	0.905596039	6.70E-05								
6102_at	NM_006915	6102	RP2	0.903978575	0.000343827								
132014_at	NM_153482	132014	IL17RE	0.901769792	0.000359429								
2745_at	NM_002064	2745	GLRX	0.901748856	0.017335081	ENSG00000173221	1	0	1				
83990_at	NM_032043	83990	BRIP1	0.894840604	0.000252584								
64343_at	NM_022461	64343	AZI2	0.892917197	5.02E-05	ENSG00000163512	2	0	1				
10993_at	NM_006843	10993	SDS	0.88942399	3.79E-05								
1612_at	NM_004938	1612	DAPK1	0.884045419	0.000457542								
54936_at	NM_017825	54936	ADPRHL2	0.882033095	5.63E-05								
23077_at	NM_015057	23077	MYCBP2	0.880254114	0.001197995								
162461_at	NM_153229	162461	TMEM92	0.879269059	7.25E-06								
2595_at	NM_198141	2595	GANC	0.879105907	0.000177662								
817_at	NM_172129	817	CAMK2D	0.877461229	0.000255516								
604_at	NM_138931	604	BCL6	0.87591552	3.12E-05	ENSG00000113916	1	1	0				
115004_at	NM_138441	115004	MB21D1	0.875271109	0.007442382								
5721_at	NM_002818	5721	PSME2	0.874400363	0.000381741	ENSG00000100911	2	1	0				
1806_at	NM_001160301	1806	DPYD	0.872242376	5.61E-05								
114991_at	NM_133374	114991	ZNF618	0.870958388	5.13E-05								
9711_at	NM_001145642	9711	KIAA0226	0.862725893	5.01E-05	ENSG00000145016	2	1	0	GCAAACGCTGCAGTGACGGT	GCAAAGCCGCTCCAAGCCAC	NM_001145642.2	1
84674_at	NM_032587	84674	CARD6	0.860107006	0.000376706								
2533_at	NM_199335	2533	FYB	0.859352836	5.14E-05								
92815_at	NM_033445	92815	HIST3H2A	0.857219238	0.003264637	ENSG00000181218	1	0	0				
3601_at	NM_172200	3601	IL15RA	0.856969076	8.99E-06	ENSG00000134470	6	3	1				
84955_at	NM_032869	84955	NUDCD1	0.852536852	0.004017842								
7162_at	NM_006670	7162	TPBG	0.850090056	4.83E-06								
28234_at	NM_019844	28234	SLCO1B3	0.847853602	0.032017224								

54149_at	NM_017447	54149	C21orf91	0.847033425	0.00243359								
4192_at	NM_002391	4192	MDK	0.844378154	0.040252807								
23258_at	NM_015213	23258	DENND5A	0.841651036	3.96E-05								
79986_at	NM_024924	79986	ZNF702P	0.840512533	0.004402211								
5129_at	NM_212503	5129	CDK18	0.838731808	0.002310237								
167127_at	NM_174914	167127	UGT3A2	0.838543002	7.19E-05								
375287_at	NM_198557	375287	RBM43	0.838028108	0.01684622								
10723_at	NM_006598	10723	SLC12A7	0.834220255	3.29E-05								
6314_at	NM_001177387	6314	ATXN7	0.831669547	0.000169255	ENSG00000163635	1	0	0				
54877_at	NM_017742	54877	ZCCHC2	0.81983518	0.001227346	ENSG00000141664	1	0	1				
2564_at	NM_021990	2564	GABRE	0.817014252	0.000559744								
3105_at	NM_002116	3105	HLA-A	0.816232693	0.000253777	ENSG00000206503	3	1	0				
54726_at	NM_199324	54726	OTUD4	0.812625117	0.028273824	ENSG00000164164	1	1	0				
56259_at	NM_030877	56259	CTNNBL1	0.811431985	1.34E-05								
23549_at	NM_012100	23549	DNPEP	0.810183924	1.94E-05								
80853_at	NM_030647	80853	JHDM1D	0.807884766	0.01065807								
54467_at	NM_019004	54467	ANKIB1	0.80480315	0.000588389								
717_at	NM_001178063	717	C2	0.803121133	0.006910371	ENSG00000166278	0	1	0				
10241_at	NM_005831	10241	CALCOCO2	0.800695038	0.003642883	ENSG00000136436	0	1	0				
329_at	NM_001166	329	BIRC2	0.795637023	0.000210076	ENSG00000110330	1	1	0				
652995_at				0.793886097	0.000381741								
128312_at	NM_175055	128312	HIST3H2BB	0.793259565	0.005560054								
9263_at	NM_004760	9263	STK17A	0.792890591	4.48E-05								
2787_at	NM_005274	2787	GNG5	0.789848622	0.000105126								
5349_at	NM_021910	5349	FXYD3	0.789620727	0.000893925								
215_at	NM_000033	215	ABCD1	0.789205402	0.000732605								
90102_at	NM_145753	90102	PHLDB2	0.786411736	0.001061886								
26037_at	NM_015556	26037	SIPA1L1	0.785039901	0.002344111								
284996_at	NM_173647	284996	RNF149	0.783917703	0.001144113								
10818_at	NM_006654	10818	FRS2	0.78037143	0.00072822								
6373_at	NM_005409	6373	CXCL11	0.780204977	0.004714163					CGCTGTCTTTGCATAGGCCCTGG	GCCTTGCTTGCTTCGATTTGGGA	NM_005409.4	1
148932_at	NM_201403	148932	MOB3C	0.77865859	0.00034431								
1520_at	NM_004079	1520	CTSS	0.777800523	0.003847116	ENSG00000163131	1	0	0				
6792_at	NM_003159	6792	CDKL5	0.777012163	0.000547331	ENSG00000008086	1	0	0				
55236_at	NM_018227	55236	UBA6	0.776952898	0.001730889								
55304_at	NM_018327	55304	SPTLC3	0.776093709	0.014401597								
54899_at	NM_017771	54899	PXK	0.77344453	0.022664111								
10410_at	NM_021034	10410	IFITM3	0.772891586	0.000678919	ENSG00000142089	4	0	2	GGTCTTCGCTGGACACCATGAATC	CGGTCACGTCGCCAACCATC	NM_021034.2	1
56985_at	NM_020233	56985	C17orf48	0.771790384	0.003323872								
57493_at	NM_020733	57493	HEG1	0.771312695	0.005058969								
100652927_at				0.77080196	0.007901922								
6913_at	NM_152380	6913	TBX15	0.770608222	0.003703032								
10009_at	NM_006777	10009	ZBTB33	0.770523566	0.000332623								
11120_at	NM_078476	11120	BTN2A1	0.765097141	0.000567524								
5993_at	NM_001025603	5993	RFX5	0.764281833	3.12E-05								
9961_at	NM_017458	9961	MVP	0.764071191	0.000213613	ENSG00000013364	1	1	0				
11221_at	NM_144729	11221	DUSP10	0.762057244	0.001968113	ENSG00000143507	0	1	0				
91056_at	NM_138368	91056	AP5B1	0.7618465	0.000151396								
143_at	NM_006437	143	PARP4	0.761379906	0.000541719	ENSG00000102699	2	1	0				
623_at	NM_000710	623	BDKRB1	0.760946	0.001336128								
219988_at	NM_152716	219988	PATL1	0.760329521	0.001301107								
7290_at	NM_003325	7290	HIRA	0.759515828	0.000116948	ENSG00000100084	1	1	0				
1844_at	NM_004418	1844	DUSP2	0.758511762	0.000756878								
113452_at	NM_033504	113452	TMEM54	0.757538936	8.58E-05								
4277_at	NM_005931	4277	MICB	0.757055422	0.000121571								
64421_at	NM_022487	64421	DCLRE1C	0.755459561	0.00022544								
9746_at	NM_014718	9746	CLSTN3	0.753440147	0.002767815								
1486_at	NM_004388	1486	CTBS	0.74953436	0.000187369								
89866_at	NM_033127	89866	SEC16B	0.748791383	7.44E-05								
7716_at	NM_007146	7716	VEZF1	0.74688264	1.31E-05								
5166_at	NM_002612	5166	PDK4	0.745371492	0.001244544	ENSG00000004799	0	1	0				
9958_at	NM_006313	9958	USP15	0.743944037	0.017368309								
64121_at	NM_022157	64121	RRAGC	0.743135543	0.00320415								
80174_at	NM_145663	80174	DBF4B	0.740767073	0.000231572								
8775_at	NM_003827	8775	NAPA	0.738957181	2.02E-05	ENSG00000105402	4	1	1				
7695_at	NM_003437	7695	ZNF136	0.738587134	0.010514194								
64651_at	NM_033027	64651	CSRNP1	0.736362521	0.000880832								
4217_at	NM_005923	4217	MAP3K5	0.735794651	0.000160716								
8528_at	NM_004032	8528	DDO	0.73546433	0.001724025								
5874_at	NM_004163	5874	RAB27B	0.734706745	0.000310991								
1906_at	NM_001955	1906	EDN1	0.734550579	0.007136857	ENSG00000078401	0	1	0				
147645_at	NM_001163922	147645	VSIG10L	0.734548013	0.002663683								
23607_at	NM_012120	23607	CD2AP	0.73005714	0.001386401								
3726_at	NM_002229	3726	JUNB	0.725712907	0.000556848	ENSG00000171223	1	1	0				
5128_at	NM_002595	5128	CDK17	0.72511598	0.003571549	ENSG00000059758	2	0	1				

54676_at	NM_019096	54676	GTPBP2	0.725041774	0.000231572	ENSG00000172432	1	1	0
8767_at	NM_003821	8767	RIPK2	0.723530158	0.001227346				
123720_at	NM_001080435	123720	WHAMM	0.721070441	0.000494418				
1390_at	NM_183060	1390	CREM	0.720331039	2.36E-05	ENSG00000095794	2	1	1
59342_at	NM_021626	59342	SCPEP1	0.715586788	0.001457401				
139285_at	NM_152424	139285	FAM123B	0.715254905	0.003815703				
841_at	NM_033357	841	CASP8	0.714979286	0.001504789	ENSG00000064012	1	1	0
57205_at	NM_020453	57205	ATP10D	0.714481849	0.000476806				
6352_at	NM_002985	6352	CCL5	0.714310498	6.92E-05	ENSG00000161570	3	2	0
57088_at	NM_020353	57088	PLSCR4	0.711881622	0.00011466				
8467_at	NM_003601	8467	SMARCA5	0.710781042	0.00018001				
390_at	NM_005168	390	RND3	0.709920564	0.000204835				
9517_at	NM_004863	9517	SPTLC2	0.709786661	0.002495447	ENSG00000100596	1	0	0
84132_at	NM_032172	84132	USP42	0.708474872	0.000357935	ENSG00000106346	1	0	0
55876_at	NM_018530	55876	GSDMB	0.706361439	0.031923342				
3612_at	NM_005536	3612	IMPA1	0.705462332	0.026727023				
10644_at	NM_006548	10644	IGF2BP2	0.704934018	0.000410558				
5939_at	NM_002898	5939	RBMS2	0.702549348	5.01E-05				
3759_at	NM_000891	3759	KCNJ2	0.699235211	0.000149265				
159013_at	NM_144970	159013	CXorf38	0.697656193	0.000479684				
7043_at	NM_003239	7043	TGFB3	0.694126494	0.005404283				
55647_at	NM_017817	55647	RAB20	0.69367275	0.000279337				
375790_at	NM_198576	375790	AGRN	0.692982067	0.000477443	ENSG00000188157	1	1	0
9541_at	NM_004882	9541	CIR1	0.692450854	0.00601042				
165324_at	NM_181713	165324	UBXN2A	0.691719058	0.001767473				
55748_at	NM_018235	55748	CNDP2	0.691636771	0.000217008				
5366_at	NM_021127	5366	PMAIP1	0.6911797	0.000529002	ENSG00000141682	5	2	1
3959_at	NM_005567	3959	LGALS3BP	0.688909838	3.29E-05	ENSG00000108679	9	4	2
5580_at	NM_212539	5580	PRKCD	0.688735995	0.000948664				
10793_at	NM_033548	10793	ZNF273	0.688290229	0.017229948				
1540_at	NM_015247	1540	CYLD	0.688225556	0.012376256				
2634_at	NM_004120	2634	GBP2	0.685338964	0.000432877	ENSG00000162645	8	2	0
162979_at	NM_145288	162979	ZNF296	0.68314807	0.001343905				
57590_at	NM_020830	57590	WDFY1	0.681356881	0.003872784	ENSG00000085449	2	0	1
3717_at	NM_004972	3717	JAK2	0.680577344	0.00336734	ENSG00000096968	3	1	0
4281_at	NM_033291	4281	MID1	0.679999183	0.000356623				
9314_at	NM_004235	9314	KLF4	0.6780724	0.005838173				
23338_at	NM_015288	23338	PHF15	0.67783015	0.00011466				
56477_at	NM_019846	56477	CCL28	0.675487419	0.001473417				
151963_at	NM_178496	151963	MB21D2	0.673608281	0.000456195				
81688_at	NM_030939	81688	C6orf62	0.672069452	0.000639513				
1266_at	NM_001839	1266	CNN3	0.671371893	0.000693659				
1026_at	NM_078467	1026	CDKN1A	0.671333718	0.000816226	ENSG00000124762	1	0	0
51762_at	NM_016530	51762	RAB8B	0.671103867	0.002259728				
5883_at	NM_004584	5883	RAD9A	0.670395762	5.59E-05				
494115_at	NM_019610	494115	RBMXL1	0.670014717	0.001037935				
5743_at	NM_000963	5743	PTGS2	0.668406524	0.042010595	ENSG00000073756	0	1	0
9451_at	NM_004836	9451	EIF2AK3	0.666151208	0.004529581				
200316_at	NM_145298	200316	APOBEC3F	0.664782116	0.02279167	ENSG00000128394	3	2	1
85463_at	NM_033390	85463	ZC3H12C	0.663913339	0.027987946				
7391_at	NM_207005	7391	USF1	0.662784317	0.000562044				
51742_at	NM_031371	51742	ARID4B	0.662317535	0.00831119				
4783_at	NM_005384	4783	NFIL3	0.662122866	0.004916198				
55293_at	NM_018314	55293	UEVLD	0.661830295	0.001075441				
84441_at	NM_032427	84441	MAML2	0.661814907	0.014605312				
80055_at	NM_024989	80055	PGAP1	0.660130973	0.002992982				
567_at	NM_004048	567	B2M	0.65931767	0.005861613	ENSG00000166710	4	1	1
6926_at	NM_016569	6926	TBX3	0.656961684	0.000116412	ENSG00000135111	1	1	0
8342_at	NM_003521	8342	HIST1H2BM	0.65654839	0.003993471				
26750_at	NM_012424	26750	RPS6KC1	0.656491345	0.005536151				
7099_at	NM_138556	7099	TLR4	0.654258839	0.012367083	ENSG00000136869	0	1	0
1326_at	NM_005204	1326	MAP3K8	0.647639631	0.000392694	ENSG00000107968	1	2	0
115273_at	NM_152304	115273	RAB42	0.647187812	0.008736178				
23162_at	NM_015133	23162	MAPK8IP3	0.647100729	0.00156498				
54463_at	NM_019000	54463	FAM134B	0.645389653	6.92E-05				
201895_at	NM_174921	201895	C4orf34	0.645127694	0.004086926				
4883_at	NM_024563	4883	NPR3	0.643455648	0.004529581				
5581_at	NM_005400	5581	PRKCE	0.642978517	0.000156595				
23032_at	NM_201626	23032	USP33	0.642877722	0.002663683				
116028_at	NM_152308	116028	RMI2	0.642780532	0.014908323				
8717_at	NM_153425	8717	TRADD	0.641513112	0.008130483	ENSG00000102871	1	0	0
144577_at	NM_152440	144577	C12orf66	0.641023985	0.000573117				
56929_at	NM_020177	56929	FEM1C	0.640361817	0.005363162				
9567_at	NM_004286	9567	GTPBP1	0.634568616	0.000231572	ENSG00000100226	1	1	0
50807_at	NM_018482	50807	ASAP1	0.634396032	0.019925418				

578_at	NM_001188	578	BAK1	0.631471768	0.000105284				
51100_at	NM_016009	51100	SH3GLB1	0.631418876	0.001387031	ENSG00000097033	1	0	0
957_at	NM_001249	957	ENTPD5	0.627267751	0.009456051				
22807_at	NM_016260	22807	IKZF2	0.626871425	0.005436155				
89894_at	NM_138341	89894	TMEM116	0.625906477	0.001806595				
79836_at	NM_024778	79836	LONRF3	0.625879873	0.007390508				
182_at	NM_000214	182	JAG1	0.62564407	0.000412204	ENSG00000101384	1	1	0
4486_at	NM_002447	4486	MST1R	0.622080276	0.000120786				
4792_at	NM_020529	4792	NFKBIA	0.62075999	0.000412204	ENSG00000100906	2	0	0
100128927_	NM_001137601	1E+08	ZBTB42	0.61955454	0.033175485				
8853_at	NM_003887	8853	ASAP2	0.619109783	0.002885453				
55689_at	NM_018023	55689	YEATS2	0.61811887	0.001550853				
23052_at	NM_015036	23052	ENDOD1	0.618079916	0.001144113	ENSG00000149218	2	1	1
2012_at	NM_001423	2012	EMP1	0.618059245	0.00190503				
117157_at	NM_053282	117157	SH2D1B	0.617549741	0.001302019				
7538_at	NM_003407	7538	ZFP36	0.617302052	0.001306984	ENSG00000128016	1	1	0
6764_at	NM_213618	6764	ST5	0.616325635	0.009013082				
11177_at	NM_182648	11177	BAZ1A	0.614068594	0.000245644				
843_at	NM_032977	843	CASP10	0.613988746	0.001578235	ENSG00000003400	0	1	0
25948_at	NM_015483	25948	KBTBD2	0.612768498	0.001481756				
114294_at	NM_171846	114294	LACTB	0.612308185	0.009577331	ENSG00000103642	1	0	1
81542_at	NM_030755	81542	TMX1	0.612061771	0.000155329				
3988_at	NM_001127605	3988	LIPA	0.610929855	0.000247038	ENSG00000107798	2	1	0
129685_at	NM_138572	129685	TAF8	0.609147554	0.001869169				
344887_at				0.607143764	0.009577331				
1052_at	NM_005195	1052	CEBPD	0.606543701	0.002031957	ENSG00000180733	1	1	0
7576_at	NM_006969	7576	ZNF28	0.606276901	0.015541257				
10392_at	NM_006092	10392	NOD1	0.60572745	0.000234141				
10158_at	NM_005764	10158	PDZK1IP1	0.605275286	0.001773891				
23347_at	NM_015295	23347	SMCHD1	0.60481058	0.000562044	ENSG00000101596	1	0	1
1666_at	NM_001359	1666	DECR1	0.603193852	0.000374925				
115825_at	NM_052950	115825	WDFY2	0.601958149	0.003576823				
84870_at	NM_032784	84870	RSPO3	0.601873868	0.000870772				
4810_at	NM_198270	4810	NHS	0.600848525	0.00783652				
121227_at	NM_153377	121227	LRIG3	0.600749102	0.003605674				
9118_at	NM_032727	9118	INA	0.599085139	0.013295396				
3164_at	NM_173158	3164	NR4A1	0.598621535	0.004801564				
84270_at	NM_032310	84270	C9orf89	0.597972348	0.032944238				
56951_at	NM_020199	56951	C5orf15	0.596408721	0.000200297				
6615_at	NM_005985	6615	SNAI1	0.596258588	0.006272357				
59348_at	NM_021632	59348	ZNF350	0.595117966	0.028267017				
4616_at	NM_015675	4616	GADD45B	0.594615009	0.004784473	ENSG00000099860	1	0	0
51421_at	NM_016201	51421	AMOTL2	0.594510934	0.000999753				
4582_at	NM_182741	4582	MUC1	0.59442765	0.040698889				
57801_at	NM_021170	57801	HES4	0.59385501	0.024861242	ENSG00000188290	1	0	0
9592_at	NM_004907	9592	IER2	0.592193694	0.016586459				
154091_at	NM_145176	154091	SLC2A12	0.59197776	0.000117969				
85450_at	NM_033397	85450	ITPRIP	0.590903422	0.001300036				
57561_at	NM_020801	57561	ARRDC3	0.590325705	0.002767668				
6648_at	NM_001024466	6648	SOD2	0.589874446	0.002863208				
57117_at	NM_020395	57117	INTS12	0.589112281	0.018901329				
84318_at	NM_032358	84318	CCDC77	0.588628424	0.000239548				
64699_at	NM_032401	64699	TMPRSS3	0.587784833	0.002291448				
648987_at				0.587260361	0.002314979				
375341_at	NM_198562	375341	C3orf62	0.586792471	0.01249249				
22856_at	NM_014918	22856	CHSY1	0.586670858	0.005080097				
